# Chromosome-scale genome assembly and characterization of *Saccharomycopsis schoenii*, a necrotrophic predatory yeast

**DOI:** 10.1093/g3journal/jkag067

**Published:** 2026-03-18

**Authors:** Divya Kriti, Marjan Barazandeh, Joseph Uche Ogbede, Guri Giaever, Corey Nislow

**Affiliations:** Department of Biochemistry and Molecular Biology, University of British Columbia, 2350 Health Sciences Mall, Vancouver, BC, Canada V6T 1Z3; Faculty of Pharmaceutical Sciences, University of British Columbia, 2405 Wesbrook Mall, Vancouver, BC, Canada V6T 1Z3; Faculty of Pharmaceutical Sciences, University of British Columbia, 2405 Wesbrook Mall, Vancouver, BC, Canada V6T 1Z3; Faculty of Pharmaceutical Sciences, University of British Columbia, 2405 Wesbrook Mall, Vancouver, BC, Canada V6T 1Z3; Department of Biochemistry and Molecular Biology, University of British Columbia, 2350 Health Sciences Mall, Vancouver, BC, Canada V6T 1Z3; Faculty of Pharmaceutical Sciences, University of British Columbia, 2405 Wesbrook Mall, Vancouver, BC, Canada V6T 1Z3

**Keywords:** *Saccharomycopsis schoenii*, genome assembly, predatory yeast, biocontrol, long-read sequencing, Hi-C scaffolding, mating-type locus, comparative genomics

## Abstract

The necrotrophic predatory yeast *Saccharomycopsis schoenii* preys on diverse fungal species including multidrug-resistant *Candida auris*, making it a promising candidate for fungal biocontrol. Despite growing interest in its predation mechanisms, a high-quality reference genome is lacking. We constructed a chromosome-scale genome assembly of *S. schoenii* using a hybrid approach combining Pacific Biosciences HiFi long-read sequencing with Hi-C chromatin conformation capture. The 14.3 Mb genome is resolved into 6 nuclear and 1 mitochondrial chromosome, demonstrating high contiguity and near-complete gene-space representation. Building upon this reference, we generated an evidence-guided annotation using BRAKER2 and eggNOG-mapper to identify genes and other genomic features. Interestingly, this complete assembly also reveals an expanded mating-type (*MAT*) system with multiple active copies that are co-transcribed, bypassing conventional silencing mechanisms. Furthermore, comparative genomics highlights species-specific expansions in several gene classes; including, secreted aspartic proteases and adhesins, linked to its necrotrophic lifestyle. This improved reference genome provides a crucial resource for investigating the molecular basis of fungal predation and mating-type evolution.

## Graphical Abstract

**Graphical Abstract:**
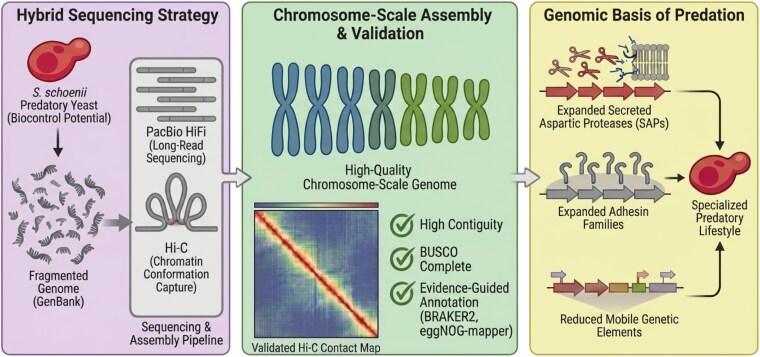

## Introduction

The yeast *Saccharomycopsis schoenii* is an aggressive, necrotrophic mycoparasite that kills and consumes other fungi. Its predation is linked to the loss of the canonical sulfate assimilation pathway, forcing the yeast to rely on organic sulfur, such as methionine derived from prey, instead of inorganic sulfate (Choo et al. 2016; Junker et al. 2019). Unlike biotrophic mycoparasites that maintain host viability, *S. schoenii* kills its prey: it detects target cells, forms penetration pegs to breach the cell wall, and consumes the cellular contents (Lachance and Pang 1997; Junker et al. 2018, 2019).

*Saccharomycopsis* is the sole genus in the family Saccharomycopsidaceae and currently comprises between 19 and 24 species (Hesselbart et al. 2018; Hajihosseinali et al. 2020; Yuan et al. 2021) spanning distinct metabolic niches, ranging from amylolytic yeasts used in food fermentations (eg *S. fibuligera, S. capsularis*) to broadly active mycoparasites (Choo et al. 2016; Karlsson et al. 2017). Phylogenetically, the genus belongs to the order Ascoideales, which includes only 2 genera: *Saccharomycopsis* and *Ascoidea* (Krassowski et al. 2018). This order utilizes a nonstandard genetic code where CUG codons are translated as serine rather than leucine. This trait is shared with the order Serinales, which comprises the classical CTG-clade pathogens, including *Candida albicans* (Krassowski et al. 2018; Groenewald et al. 2023; Ó Cinnéide et al. 2024). Despite this shared genomic feature, *S. schoenii* acts as a predator toward its distant relatives, exhibiting broad prey specificity. For example, it has been shown to kill multidrug-resistant *Candida auris* and other pathogenic *Candida* spp., as well as phytopathogenic molds such as *Penicillium digitatum, Penicillium italicum*, and *Penicillium expansum* (Pimenta et al. 2008; Junker et al. 2018). This combination of aggressive predation and broad prey range has attracted interest in using *S. schoenii* as a biocontrol agent. Such applications require a comprehensive understanding of its genomic architecture and molecular mechanisms that underlie its predation.

Detailed genomic resources for *S. schoenii,* however, remain limited. The current assembly (Junker et al. 2019) comprises 47 scaffolds with an N50 of only 793.5 kb. This fragmentation confounds the resolution of complex gene families and regulatory elements potentially linked to predation, as well as important structural features like mating-type (*MAT*) loci and centromeres. Evidence from other mycoparasites, such as *Clonostachys rosea*, demonstrates that chromosome-scale assemblies are essential for connecting expanded gene clusters (like polyketide synthases) to antagonistic phenotypes (Karlsson et al. 2015; Fatema et al. 2018). Therefore, a contiguous reference is required to resolve both the evolutionary architecture and the functional repertoire of *S. schoenii*.

Here, we generate a high-quality, chromosome-scale genome assembly of *S. schoenii* using a hybrid strategy combining Pacific Biosciences High-Fidelity (PacBio HiFi) long-read sequencing with Hi-C chromatin conformation capture. Long reads enable resolution of repetitive regions and structural variation, while Hi-C provides long-range linkage information to scaffold contigs into complete chromosomes (Lieberman-Aiden et al. 2009). This assembly allows us to resolve the *MAT* locus, define centromeric architecture, and annotate the non-coding genome, such as tRNAs and long terminal repeat (LTR) retrotransposons, providing a robust framework for dissecting the genetic basis of necrotrophic predation.

## Materials and methods

### Strain and DNA extraction

*Saccharomycopsis schoenii* strain NCYC 2975 (equivalent designation GDD12) was obtained from the National Collection of Yeast Cultures (NCYC, UK). The strain originated from apple juice (depositor A. Boyd, August 2000) and is listed with no known Nagoya Protocol restrictions. Cultures were freeze-dried and revived on YM agar/broth per NCYC guidance. High molecular weight (HMW) genomic DNA was extracted using a phenol-chloroform protocol adapted from Protocols.io method “Extraction of yeast high molecular weight genomic DNA” (Hoyer 2023). A 55 mL culture at OD 12 was harvested by centrifugation, lysed gently (no vortexing), and treated sequentially with RNase A and proteinase K.

HMW DNA was brought up in 500 µL TE, after which RNase A (5 µL) was added and the suspension was left at room temperature for 4 h to ensure complete dissolution and initial RNA removal. Proteinase K (5 µL) was then added and incubated at 50 °C for 15 min to digest residual proteins. The lysate underwent phenol:chloroform extraction (510 µL; gentle inversion, phase separation by centrifugation), and the aqueous phase was recovered and eluted in 1 mL nuclease-free water. A second RNase A treatment was performed at 37 °C for 30 min followed by phenol-chloroform and collection in 700 µL nuclease-free water. Nucleic-acid quality and yield were assessed on a Cytation 5 spectrophotometer (A260/280 = 1.87) and by fluorometry (Qubit dsDNA BR: ∼20 ng/µL); downstream input calculations used the Qubit measurement due to its specificity for double-stranded DNA. The resulting HMW DNA was assessed with Tapestation (Agilent, 5067–5366 and 5067–5365), confirming a dominant peak at ∼60 kb, indicating its suitability for long-read sequencing with PacBio (Supplementary Fig. 1). The DNA was then submitted to Canada's Michael Smith Genome Sciences Centre (BCGSC) for PacBio Revio library construction with BluePippin size selection to enrich for long inserts, and sequenced as a singleplex library on a Revio SMRT Cell.

### PacBio HiFi sequencing and de novo assembly

HiFi long reads were generated on the PacBio Revio platform using circular consensus sequencing (CCS), producing 3.7 million reads totaling 59.5 Gb of sequence data (∼4,100× theoretical coverage for a ∼15 Mb genome). Reads were exported with SMRT Link 13.1.0.221970 with built-in CCS-adapter handling. Read quality was assessed with FastQC v0.12.0 (Andrews 2010). De novo genome assembly was performed using the Improved Phased Assembler v1.8.0, executed via the pbcromwell on a Slurm HPC environment (wall time 60 h; 2 nodes; 2 tasks; 32 CPUs per task; NPROC = 128; 754 GB RAM). The run specified a 15 Mb genome size prior. Reads were downsampled to 100× to balance accuracy and compute, and assembled with phasing, polishing, and cleanup of intermediates. Assemblies generated with and without duplicate purging were compared across coverage thresholds. The duplicate-purged 100× assembly consistently outperformed the non-purged build and was used for downstream work. Phasing allowed for the resolution of heterozygosity and repeats, ensuring a clean separation of haplotypes before the final duplicate purging step, to produce the final primary assembly. Assembly quality control used QUality ASsessment Tool or QUAST v.5.2.0 to summarize contiguity metrics (eg N50, L50, total size) and Benchmarking Universal Single-Copy Orthologs or BUSCO v5.4.7 (lineage set: saccharomycetes_odb10) to assess completeness (Gurevich et al. 2013; Manni et al. 2021; Tegenfeldt et al. 2025).

### Hi-C library preparation and chromatin conformation analysis

Hi-C libraries were prepared using an *in situ* Hi-C workflow. Two *S. schoenii* cultures were grown in yeast extract-peptone-dextrose for 48 h, harvested by centrifugation, resuspended in phosphate-buffered Saline (PBS) to OD 1.0, and crosslinked with 1% (v/v) formaldehyde for 20 min at room temperature with intermittent mixing, after which glycine was added to a final concentration of 125 mM to quench crosslinking for 15 min. Cells were harvested (10,000 × g, 5 min) and washed in PBS, and frozen prior to shipment to Phase Genomics (https://phasegenomics.com/) for library preparation and sequencing. From these crosslinked pellets, Hi-C libraries were prepared using the Proximo Hi-C Kit (Fungal) v4.5 according to the manufacturer's protocol (https://info.phasegenomics.com/hubfs/Protocol_Fungal_v4.5_202404.pdf). These crosslinked cells were lysed and chromatin was immobilized on magnetic beads, fragmented enzymatically (DpnII), and subjected to end repair with incorporation of biotin-labeled nucleotides. Proximity ligation was then carried out under dilute conditions to join DNA fragments that were spatially proximal in the nucleus (Lieberman-Aiden et al. 2009). After ligation, crosslinks were reversed at 65 °C, and proteins digested to release ligation products. DNA was purified, biotin-labeled ligation junctions were captured on streptavidin-coated magnetic beads, and Illumina-compatible sequencing libraries were constructed on-bead, including adapter ligation, PCR amplification, and size selection. Library fragment size and concentration were determined using TapeStation and quantitative PCR (qPCR), and library performance was evaluated by low-pass sequencing (∼200,000 read pairs on an Illumina iSeq System). The Hi-C libraries passed these QC steps and were then sequenced (NovaSeq X Plus 10B) as 150 bp paired-end reads.

### Chromosome-scale scaffolding and curation

Hi-C reads were aligned to the duplicate-purged 100 × assembly using BWA-MEM v0.7.18 (Li 2013), with alignment filtering and coordinate sorting performed using SAMtools v1.19.2 (Li et al. 2009). Alignment quality and Hi-C library metrics were assessed with hicQC v3.7.2 (HiCExplorer v3; Wolff et al. 2020) to evaluate valid pair proportions, contact density distributions, and coverage uniformity prior to scaffolding. Chromosome-level scaffolding was executed using YaHS v1.2.2 (Zhou et al. 2023), which uses Hi-C contact frequency matrices to order and orient contigs. The resulting scaffolds were manually inspected Juicebox (GUI) v2.17.00 (Durand et al. 2016) and refined in Juicebox Assembly Tools (JBAT) (Dudchenko et al. 2018) to correct mis-joins, optimize orientations, and identify potential structural inconsistencies. Iterative rounds of YaHS scaffolding followed by manual JBAT curation progressively improved assembly contiguity and completeness, tracked using N50, auN, and BUSCO metrics. Hi-C contact maps visualized in Juicebox confirmed contiguous chromosomal organization, centromere placement, and enabled telomere-to-telomere validation of scaffold ends. The resulting chromosome-scale assembly of *S. schoenii* has been deposited in NCBI GenBank under accession JBTFFQ000000000 (Version JBTFFQ010000000; BioProject PRJNA1364152).

### Evidence-guided annotation

**Fig. 1.**
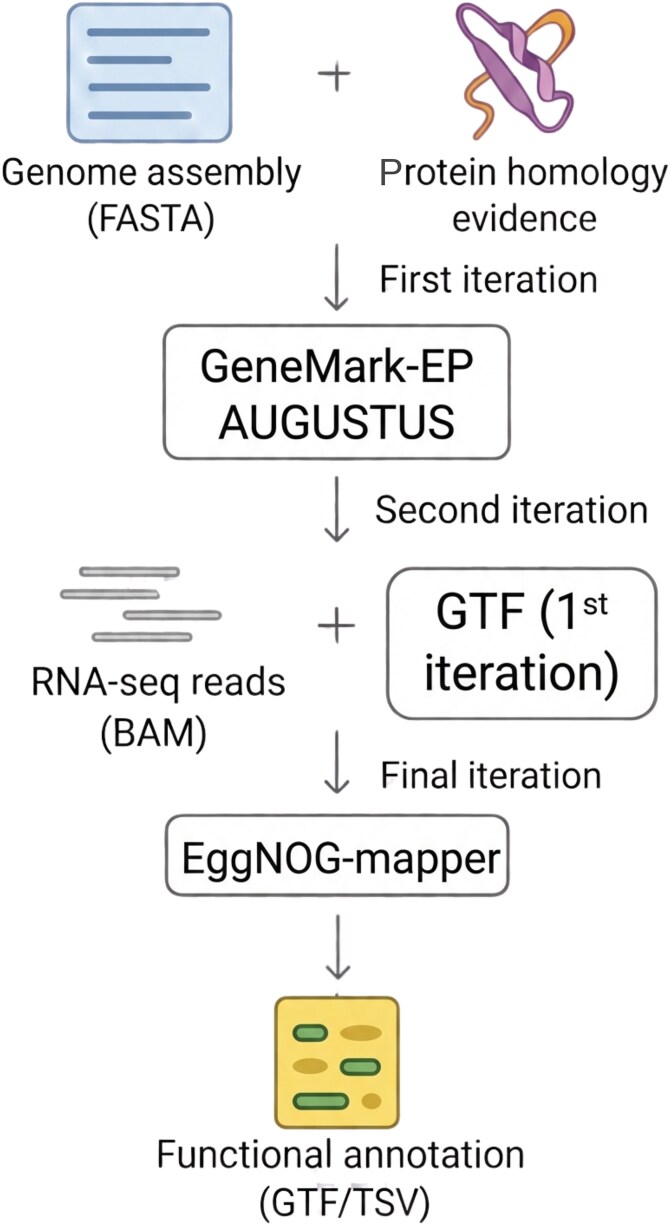
Evidence-guided approach for *S. schoenii* genome annotation. Protein homology and RNA-seq data were sequentially integrated with GeneMark-EP, BRAKER2, and AUGUSTUS to improve gene predictions over ab initio models. Functional annotation of predicted genes was performed using EggNOG-mapper, generating the final GTF annotation file. Created in BioRender. Kriti, D. (https://BioRender.com/gv2075n) is licensed under CC BY 4.0.

Gene annotation for the *S. schoenii* genome was performed using an evidence-guided pipeline that integrated both protein homology and RNA-seq data to improve prediction accuracy relative to *ab initio* approaches alone (Fig. 1). Protein-supported gene prediction was first carried out with GeneMark-EP (Brůna et al. 2020), which leveraged homologous protein sequences from related fungal ascomycete species (*C. albicans*, *C. auris*, and *S. cerevisiae*) to identify coding regions and generate preliminary gene models. To get expression data, total RNA was extracted in triplicate using a one-step hot formamide protocol, and non-stranded poly(A)-enriched mRNA libraries were sequenced as 150 bp paired-end reads (∼50 million pairs per sample; Illumina). These RNA-seq reads were aligned to the genome using the splice-aware aligner STAR v2.7.11b (Dobin et al. 2013), and the resulting BAM files were supplied to BRAKER2 v2.1.6 (Brůna et al. 2021) to extract intron-exon junction evidence and splice-site hints. BRAKER2 combined the RNA-seq evidence with GeneMark-EP output to train and refine AUGUSTUS v3.4 (Stanke et al. 2008) gene models. The –fungus and –softmasking parameters were used to optimize for fungal gene structures and correctly handle repetitive regions. Functional annotation of the predicted protein-coding genes was performed with EggNOG-mapper (Cantalapiedra et al. 2021) using –itype proteins option, which assigned putative gene names, functional categories, Gene Ontology (GO) terms (Ashburner et al. 2000; The Gene Ontology Consortium, 2026), KEGG pathway annotations (Kanehisa et al. 2023), and orthologous group information based on sequence similarity and orthology relationships to curated reference databases. The final annotation file is available at Supplementary File 1.

For intron analysis and characterization, genome coordinates were extracted from the final GTF annotation. To validate consensus splice sites, genomic sequences corresponding to intron start and ends were extracted from the *S. schoenii* genome assembly using custom Python scripts. The 5′ and 3′ terminal dinucleotides were assessed for conformity to the canonical GT-AG consensus. Intron length statistics (mean, median, min/max) were calculated across all 708 intron-containing genes. The annotated gene set for conserved families known to retain introns in Saccharomycotina, were also analyzed using custom scripts. The distribution of intron lengths was visualized using Matplotlib. The custom scripts used for intron extraction and motif validation are available at GitHub (https://github.com/dkriti/Saccharomycopsis-schoenii-Genome-Assembly).

### Genomic architecture

Transfer RNA (tRNA) genes were annotated using tRNAscan-SE v2.0.12 (Chan et al. 2021) with eukaryotic parameters (-E). The identified loci were filtered to ensure coverage of all 20 standard amino acids. To identify Transposable Elements (TEs), specifically LTR retrotransposons, the LTRharvest tool (Ellinghaus et al. 2008) from the GenomeTools suite v1.6.2 (Gremme et al. 2013) was used. A suffix array index for the repeat-masked *S. schoenii* assembly was generated using gt suffixerator with options -tis -suf -lcp -des -ssp -sds -dna. LTR candidates were then identified with gt ltrharvest using a minimum LTR length of 100 bp, maximum LTR length of 1,000 bp, minimum LTR distance of 1,000 bp, maximum distance of 15,000 bp, and minimum LTR similarity of 85% (which allowed for the detection of both intact elements and slightly diverged remnants). Evolutionary classification of the extracted LTR retrotransposons was performed using TEsorter v1.5.1 (Zhang et al. 2022). To achieve that, the nucleotide sequences of these identified elements were first extracted from the genome assembly using the getfasta utility of bedtools v2.31.0 (Quinlan and Hall 2010) followed by protein domain profiling and classification. TEsorter was executed using the Gypsy Database (GyDB; Llorens et al. 2011) to ensure accurate lineage resolution for fungal retrotransposons. Elements were classified into superfamilies (*e.g. Ty1/*Copia or *Ty3/*Gypsy) and specific evolutionary clades based on the detection and ordering of conserved retroviral protein domains, specifically reverse transcriptase (RT), integrase (INT), RNase H, and aspartic protease (AP). Elements exhibiting domain matches across multiple reference lineages were designated as “mixed.” The physical size and structure of each candidate element were analyzed using the coordinate data from LTRharvest. The total length of each element, as well as the size of its LTR ends, were measured to separate standard, potentially active transposons from unusually large ones. Such large elements typically represent nested insertions where 1 transposon has integrated inside another. To estimate how recently an element integrated into the genome, the sequence similarity between the paired 5′ and 3′ LTRs of each full-length element was compared. Because paired LTRs are identical at the time of insertion, their similarity acts as an evolutionary clock. Finally, extremely long elements (>10 kb) that lacked recognizable protein domains were classified as disrupted nested insertions.

Centromeric loci were initially identified by manually inspecting chromatin conformation capture (Hi-C) contact maps using Juicebox to locate regions with strong 3D clustering (Rabl configuration). To assess the nature of these loci, they were screened for the conserved *S. cerevisiae* CDEI-spacer-CDEIII kinetochore-binding motif to test for the presence of point centromeres using custom Python scripts. Following this motif analysis, the boundaries of the regional centromeres were defined by analyzing the genome assembly for heterochromatic AT-bias. A sliding-window nucleotide composition analysis was performed, and the highest non-overlapping AT-rich peak per chromosome was isolated. The coordinates of these AT-islands were used to extract the complete centromeric core sequences or downstream analysis. The presence of conserved flanking housekeeping genes was confirmed using a local synteny script. To determine the centromeric type, the exact genomic boundaries of the centromeres were evaluated against the total chromosome lengths. The long-arm (q) to short-arm (p) physical distances were calculated for each chromosome, and morphological types were assigned according to the standard Levan classification system arm ratios (Levan et al. 1964). TEs within the extracted centromere cores were annotated using both homology-based and de novo structural methods. Homology searches were performed using RepeatMasker (v4.2.2; Saha et al. 2008) with the NCBI/RMBLAST engine and the Dfam v3.9 fungal database (parameters: -pa 4 -species fungi -gff; Storer et al. 2021). For structural de novo identification, the GenomeTools suite was used to build a suffix array (gt suffixerator -tis -suf -lcp -des -ssp -sds -dna). LTRs were identified using LTRharvest (parameters: -minlenltr 100 -maxlenltr 1,000 -mintsd 4 -maxtsd 20 -motif tgca -motifmis 1 -vic 10 -seed 76 -seqids yes -similar 85). The resulting LTR elements were functionally classified and screened for conserved retrotransposon protein domains using TEsorter (GyDB). Following the exclusion of intact TEs, the cores were analyzed for uncharacterized centromeric satellite DNA arrays using Tandem Repeat Finder (TRF; Benson 1999). To filter out random AT-rich background noise and isolate biologically conserved repeating units, TRF was executed with stringent parameters (match: 2, mismatch: 7, delta: 7, matching probability: 80, indel probability: 10, minscore: 50, maxPeriod: 2000). To analyze sequence conservation across all 6 centromeres, the dominant consensus sequence from each chromosomal core was extracted and structurally aligned using MUSCLE v3.8.1551 (muscle -align; Edgar 2004). The multiple sequence alignment was visualized as a sequence logo via WebLogo 3 (parameters: -D fasta -F pdf -A dna –color-scheme classic; Crooks et al. 2004), allowing for the calculation of positional Shannon entropy (bits in *y*-axis) to determine structural conservation. To visualize the satellite arrays within their chromosomal context, the localized tandem repeat coordinates were mapped back to their whole-genome coordinates and visualized using the Integrative Genomics Viewer v2.9.1 (IGV; Thorvaldsdóttir et al. 2013) to assess the spatial relationship between the repeat-dense centromere cores, housekeeping genes, and the chromosome itself.

To characterize the genomic architecture of the *MAT* loci in *S. schoenii*, local alignment searches were performed against the chromosome-scale reference assembly using tblastn (blast 2.16.0; Camacho et al. 2009). Query sequences consisted *S. cerevisiae* and *C. albicans* orthologs for the mating-type transcription factors (*MATa1, MATa2, MATɑ1, MATɑ2*) as well as the anchor genes (*SLA2* or Src-like adaptor 2 *and DIC1* or dicarboxylate carrier 1). Significant hits were filtered to define the exact genomic boundaries of the *MAT* cassettes across all 6 chromosomes. They were structurally annotated and visualized using SnapGene software (www.snapgene.com) v8.2.2. To resolve unannotated transcriptional peaks near centromeric boundaries, translated nucleotide queries via BLASTx (blast 2.16.0; Camacho et al. 2009) were executed against the NCBI non-redundant (nr) protein database. Transcriptional activity across the expanded *MAT* loci was assessed using RNA-seq data derived from *S. schoenii* (3 biological replicates). RNA-seq reads were aligned to the *S. schoenii* reference genome using the STAR aligner v2.7.11b (Dobin et al. 2013) and the alignments and read coverage were visualized using IGV. Custom scripts were used to cross-reference RNA-seq splice junctions with the mapped genomic boundaries of the *MAT* orthologs. Exact splice junction coordinates and read support metrics were extracted natively from the STAR aligner's SJ.out.tab output files. Following this, the start and end coordinates of these introns were compared against the nearest upstream (5′) and downstream (3′) BLAST hit boundaries of the *MAT* and *SLA2* open reading frames (ORFs). To evaluate the spatial distribution of the *MAT* cassettes, the physical distance of each locus to its nearest centromere and telomere was calculated. Like the centromere, telomeric boundaries were also extracted directly from the Hi-C contact map assembly data using Juicebox. The *SLA2-MAT* cassettes, centromeres and their flanking pericentromeric space were analyzed using tRNAscan-SE with Eukaryotic search mode (-E) to detect potential tRNA genes. To investigate the structural mechanism underlying homothallic switching, the nuclear chromosomes were analyzed for the presence of inverted repeats using Inverted Repeats Finder v3.09 (IRF; Warburton et al. 2004). Parameters included match: 2, mismatch: 3, delta: 5, match probability: 80, indel probability: 10, minscore: 40, max distance: 50,000, max length: 10,000).

### Comparative genomic analysis

A comparative analysis was performed against 4 different yeasts: *Saccharomycopsis fibuligera* KPH12, *S. cerevisiae* S288C, *C. albicans* SC5314, and *C. auris* B8441 (a clinically relevant strain). Orthologous gene clusters were identified using OrthoVenn3 (Sun et al. 2023) with default parameters (*E*-value threshold 1e-5, inflation value 1.5). OrthoVenn3 employs OrthoFinder for ortholog inference (Emms and Kelly 2019) and CAFE5 for gene family expansion/contraction analysis along the inferred species tree (Mendes et al. 2021). The species phylogeny was reconstructed using maximum likelihood with the JTT+CAT substitution model based on concatenated alignments of single-copy orthologs (Jones et al. 1992). Gene family evolution estimated birth-death rates of gene families along phylogenetic branches and identified statistically significant expansions and contractions. Functional annotation of these expanded and contracted gene families was performed using GO enrichment analysis (Subramanian et al. 2005) within OrthoVenn3, with significance thresholds of *P* < 0.05. Sulfate assimilation pathway loss was verified by BLASTp (*E*-value threshold 1 × 10^−5^; Camacho et al. 2009).

## Results and discussion

The *S. schoenii* genome was resolved into a high-quality, chromosome-scale assembly that provides a reference for functional and evolutionary analyses. PacBio long-read sequencing generated approximately 3.7 million reads from ∼60 kb DNA molecules, yielding 59.5 billion bases at a deep ∼4,100× raw coverage of the *S. schoenii* genome.

### Genome assembly, scaffolding, and quality

**Fig. 2.**
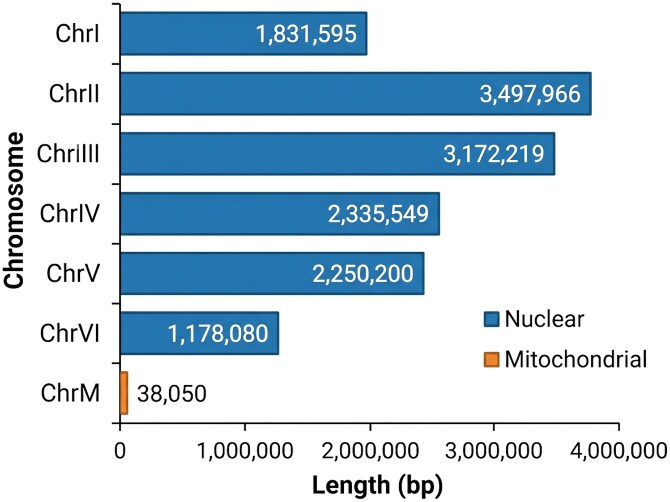
Chromosome-level statistics from the *Saccharomycopsis schoenii* genome assembly. Six nuclear chromosomes range in size from ∼1.2 to 3.5 Mb. The mitochondrial genome was identified based on its small size (∼38 kb) and its circular topology.

The hybrid PacBio + Hi-C assembly produced a contiguous chromosome-scale genome of *S. schoenii* of 14.3 Mb, consistent with genome sizes reported for closely related *Saccharomycopsis* species and other Saccharomycetales yeasts (Junker et al. 2019). Detailed Hi-C library QC metrics are provided in Supplementary Table 1. The initial PacBio assembly of 13 contigs was scaffolded using Hi-C contact data and refined through manual curation with JBAT to generate 7 chromosome-scale scaffolds totaling 14.3 Mb with 33.9% GC content. Contiguity metrics indicated that the largest scaffold was 3,497,966 bp; QUAST reported N50 = 2,335,549 bp, N90 = 1,831,595 bp, L50 = 3, and L90 = 5. The auN statistic, which measures the average length of the DNA sequence containing a randomly selected base, was 2,625,970.7 indicating that most bases are found in very large, contiguous DNA segments. This high auN value supports the N50 metric (Li, 2020). Gap content was very low at 0.003% (3.12 Ns per 100 kb), indicating minimal scaffold joins through unknown sequences and supporting the structural integrity of the assembly (Table 1). The 7 scaffolds comprised 6 nuclear chromosomes (ChrI-VI; 1.2 to 3.5 Mb) and 1 mitochondrial chromosome (ChrM; 38 kb), identified based on its size and circular topology (Fig. 2).

**Table 1.** Key statistics of the *Saccharomycopsis schoenii* genome assembly.

A. QUAST summary (contiguity and size)		B. BUSCO summary (quality and completeness)	
Assembly size	14,303,659 bp	GC content	33.9%
Scaffolds/contigs	7/13	N's per 100 kb	3.1
Largest contig	3,497,966 bp	BUSCO (saccharomycetes_odb10)—Complete (C)	93.0% (1,988)
N50	2,335,549 bp	BUSCO—single-copy (S)	92.2% (1,970)
N90	1,831,595 bp	BUSCO—duplicated (D)	0.8% (18)
L50/L90	3/5	BUSCO—fragmented (F)	1.5% (33)
auN	2,625,970.70	BUSCO—missing (M)	5.5% (116)
Assembly gaps	0.003%	BUSCO—total groups (*n*)	2,137

Assembly completeness and gene-space representation (the proportion of conserved genes captured in the assembly) were evaluated using BUSCO with the saccharomycetes_odb10 dataset. The final assembly contained 1,988 complete BUSCOs (93.0%), of which 1,970 (92.2%) were single-copy and 18 (0.8%) duplicated, together with 33 fragmented (1.5%) and 116 missing (5.5%) loci out of 2,137 groups searched (Table 1). These 1,988 complete BUSCOs represent conserved single-copy orthologous genes across the Saccharomycetes lineage and indicate that the assembly captures the vast majority of the conserved gene content with minimal duplication, consistent with a haploid, high-completeness primary assembly. Collectively, these metrics show that the *S. schoenii* genome sequence is contiguous, nearly complete, and suitable as a reference for downstream analyses, such as transcriptomics, fitness profiling, and comparative genomics.

### Hi-C contact maps and chromosome-scale validation

**Fig. 3.**
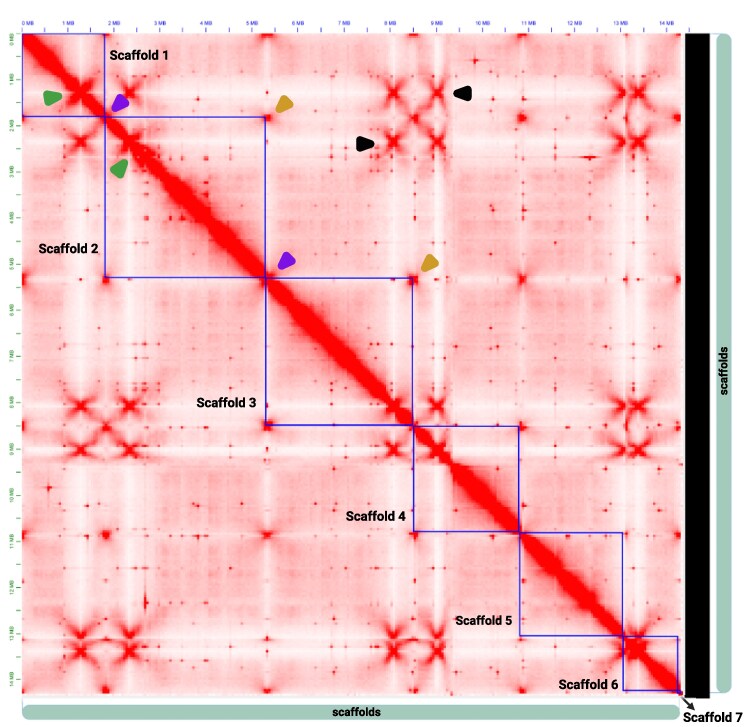
Hi-C contact map validates chromosome-scale scaffolds. The axes represent the assembled *S. schoenii* genome laid out end-to-end; color intensity indicates chromatin contact density. Blue boxes mark the 7 chromosome-scale scaffolds (scaffold 1 to 7). The bright main diagonal within each box shows strong intra-chromosomal contacts with no breaks or mis-joins. *Intra-chromosomal features:* Green arrowheads indicate centromere positions (high-intensity interaction hubs). Purple arrowheads mark telomeric regions at scaffold boundaries. *Inter-chromosomal architecture:* Black arrowheads point to centromere-centromere clustering (off-diagonal focal interactions). Gold arrowheads indicate telomere-telomere clustering (grid-like interactions at scaffold intersections), validating correct chromosome orientation and complete chromosome ends.

Hi-C scaffolding and contact map analysis were used to validate the chromosome-scale organization of the assembly and confirm scaffold orientation and contiguity (Fig. 3). The Hi-C contact map provides a visual representation of spatial proximity within the genome, with both axes representing the assembled *S. schoenii* scaffolds laid out end-to-end and color intensity indicating contact density. A broad, continuous main diagonal within each scaffold (outlined with blue boxes) indicates strong within-scaffold contacts and the absence of obvious breaks or mis-joins, consistent with correctly assembled chromosome-length units. Distinct boundaries between diagonals marked the separation of individual chromosomes, while a low level of off-diagonal inter-scaffold contacts supports the lack of major chimeric joins.

The contact map revealed bright cross-scaffold interaction bands corresponding to centromere clustering (marked by large stars in Fig. 3). Enrichment of contacts between scaffold termini (marked by small stars) showed telomere-telomere interaction foci. No evidence of terminal mis-joins was detected, supporting the correct placement of chromosome ends and telomere-to-telomere completeness. Together, the centromere and telomere interaction patterns are characteristic of a Rabl-like chromatin configuration, in which chromosomes are oriented with centromeres clustered at a single nuclear site (Rabl 1885; Duan et al. 2010), as reported in other budding yeasts. These Hi-C features support our conclusion that the final scaffolds represent intact chromosomes and that the *S. schoenii* assembly accurately reflects the native chromosomal architecture.

### Benchmarking against the existing *S. schoenii* reference

The new assembly, generated independently, was benchmarked against the existing *S. schoenii* reference genome (Junker et al. 2019; GenBank: GCA_010994365.1) (Table 2). The reference GenBank record contains 47 scaffolds, including 18 short sequences (<11.5 kb) likely representing assembly debris or mitochondrial fragments not accounted for in the original publication's report of 29 contigs. Overall, the previous reference was highly fragmented, comprising 142 underlying contigs. In contrast, our assembly significantly improved contiguity, consolidating the genome into 13 contigs scaffolded into 7 chromosome-scale units. This improvement is reflected in the metrics: the scaffold N50 increased from 793.5 kb to approximately 2.3 Mb, while the scaffold L50 decreased from 7 to 3 (Table 2).

**Table 2.** Comparison: new assembly vs previous GenBank reference.

Metric	Previous GenBank Assembly	New Assembly	Improvement
Total length (Mb)	13.8	14.3	+0.5
Number of contigs	142	13	11-fold reduction
Number of scaffolds	47	7	6.7-fold reduction
Scaffold N50 (kb)	793.5	2,335.6	2.9-fold increase
Scaffold L50	7	3	2.3-fold reduction

### Evidence-guided genome annotation

The *S. schoenii* genome was annotated using an evidence-guided pipeline that integrated *ab initio* gene prediction with protein homology and RNA-seq support (Fig. 1). This protein-homology-guided annotation identified 5,447 genes and 5,614 transcripts, dominated by single-exon ORFs (4,543 single-exon genes, 83.4%). Incorporating RNA-seq evidence modestly reduced the total gene count to 5,428 genes but substantially improved gene structure. For example, the transcript data helped remove genes that were only weakly supported and to merge predicted isoforms that did not match actual RNA evidence. In the protein-only annotation, 904 genes (16.6%) contained introns and 160 (2.9%) encoded multiple transcripts. In the final RNA-guided annotation, the number of intron-containing genes decreased to 708 (13.0%), and genes with multiple transcripts dropped to 44 (0.8%). The final annotation comprised 5,428 protein-coding genes, 5,473 transcripts, and 6,327 exons, with 4,720 single-exon genes (87.0%), capturing substantially more exonic structure (such as splice variants and extended UTRs) than the homology-only version with improved splice-site resolution.

The intron density in *S. schoenii* is low among the Saccharomycetes. With 708 genes containing introns (13%) and an average of 0.17 introns per gene, *S. schoenii* exhibits a higher intron incidence than *S. cerevisiae* (4%–5%; Bon et al. 2003; Hooks et al. 2014) and *C. albicans* (4%–6%; Mitrovich et al. 2007) but far below intron-rich filamentous ascomycetes (>50%) and basidiomycetes such as *Cryptococcus neoformans* (∼99%; Janbon et al. 2014). Minimal alternative splicing was observed: only 0.8% of genes produced multiple transcripts, consistent with the generally low rates of alternative splicing across the Saccharomycotina subphylum, which has undergone extensive intron loss during evolution (Stajich et al. 2007). These features place *S. schoenii* in an intermediate position among budding yeasts, with largely compact gene models and sparse introns, yet with slightly more introns than the most intron-poor yeast.

We sought to characterize these introns further. Structural annotation, guided by RNA-seq evidence, identified 884 introns within the gene set. Analysis of these gene models confirmed that 100% of the introns conform to the canonical GT-AG splice site consensus. The intron lengths range from 37 to 2,318 bp, with a mean of 259.8 bp and a median of 113.0 bp (Supplementary Fig. 2). This distribution is characteristic of Saccharomycotina, where the mean is skewed by a subset of genes harboring exceptionally long introns (Neuvéglise et al. 2011). Furthermore, introns were predominantly found in the highly expressed, conserved gene families, including actin (*ACT1*), translation elongation factors (*TEF1, EFB1*), and ribosomal protein genes (eg *RPL7, RPS6*). Beyond these usual suspects, we also identified additional conserved regulatory genes with large introns as shown in Supplementary Table 3. Notable examples include the yeast RNA annealing protein *YRA1* (containing introns of 850 and 748 bp) and the DEAD-box RNA helicase *DBP2* (1,393 bp intron). These long introns are known to play crucial roles in autoregulation and stress response in related yeasts (Parenteau et al. 2019). In contrast, most of the genome, including many vacuolar ATPase (*VMA*) subunits, have shorter introns, with sizes close to the 113 bp median.

Functional annotation using EggNOG-mapper assigned at least 1 functional attribute (homology-based gene name, GO term, and/or COG category) to ∼85% of genes in the final GTF, with the remaining ∼15% lacking recognizable homologs or ontology assignments. The high proportion of annotated genes supports both the completeness and evolutionary conservation of the gene set. The unannotated ORFs likely correspond to species- or genus-specific genes, rapidly evolving proteins, or functions underrepresented in current databases. The overall gene count of ∼5,400 genes is in line with expectations for Saccharomycetales yeasts, which typically harbor 5,000 to 6,000 protein-coding genes (Goffeau et al. 1996). Together, the compact gene models, low intron frequency, and sparse alternative splicing are characteristic of budding yeast genomes and indicate that the final *S. schoenii* annotation is both accurate and representative.

### Genome architecture and non-coding elements

#### tRNA repertoire

The characterization of the non-coding and repetitive element landscape was also performed to offer a comprehensive view of the *S. schoenii* genome architecture beyond the protein-coding space. A total of 160 tRNA loci were identified, comprising 140 nuclear and 20 mitochondrial genes (Supplementary File 3). *S. schoenii* encodes nuclear tRNAs for all 20 canonical amino acids, confirming the capacity to decode the full standard amino acid repertoire. The total nuclear count (140 genes) is consistent with other species in the Saccharomycotina that have not undergone Whole Genome Duplication (WGD), such as *Kluyveromyces lactis* (162 genes) and *C. albicans* (130 genes), and contrasts with the expanded set found in the WGD yeast *S. cerevisiae* (275 genes) (Dujon et al. 2004; d’Enfert 2004; Chan and Lowe 2016 [https://gtrnadb.org/genomes/eukaryota/Scere3]). The redundancy of specific isotypes follows the wobble-base pairing rules typical of the lineage (Bloom-Ackermann et al. 2014). For example, Arg-TCT and Lys-CTT isotypes are highly expanded (Supplementary File 3), suggesting selection for translational efficiency of specific optimal codons.

**Fig. 4.**
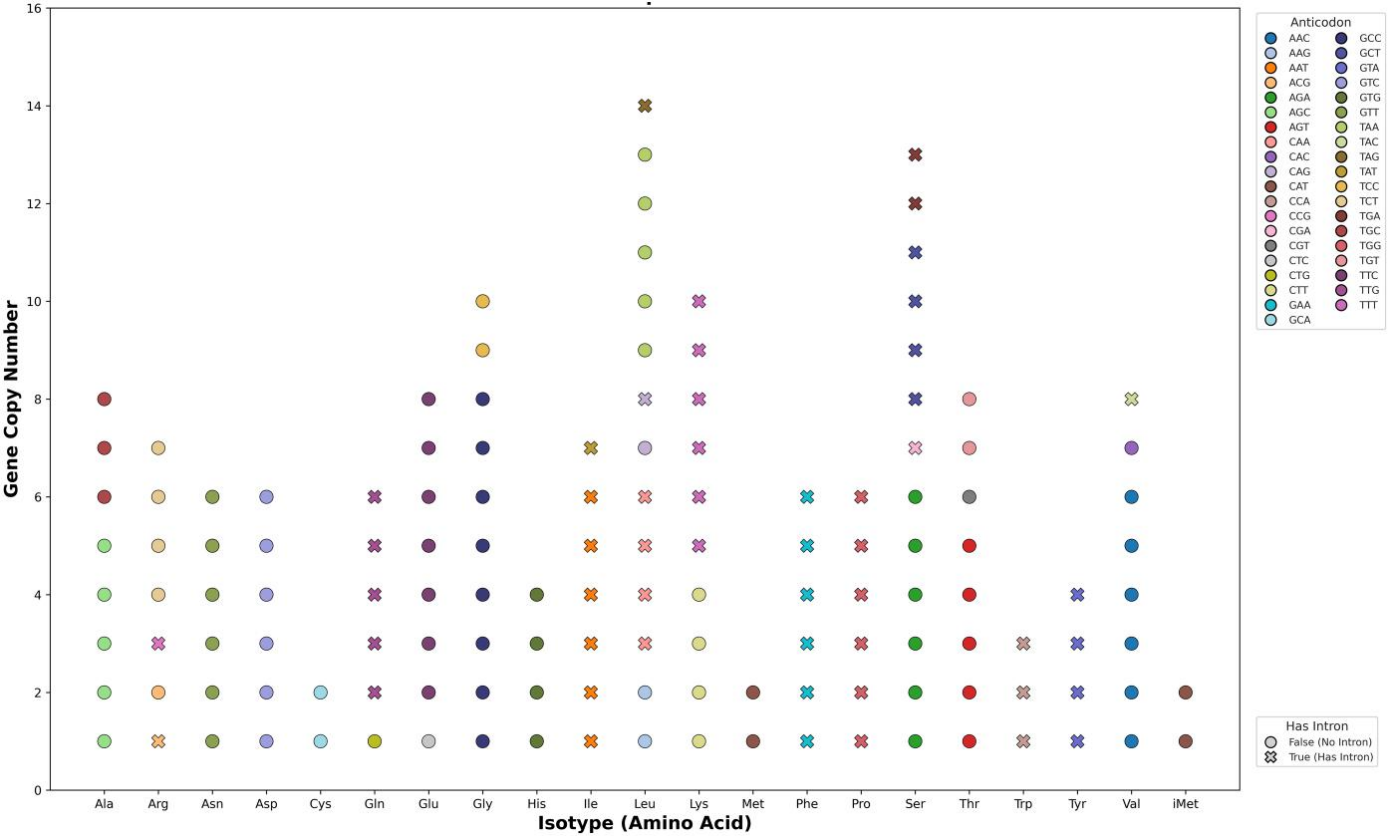
Nuclear tRNA repertoire and isodecoder diversity in *S. schoenii.* The distribution of 140 nuclear-encoded tRNA genes grouped by amino acid isotype. a) Genes are colored by their anticodon identity, representing 39 distinct nuclear isodecoders. The repertoire includes all 20 canonical amino acids plus the initiator methionine (iMet), plotted separately from elongator methionine (Met). b) Markers indicate gene structure: circles (o) represent intron-less genes, while crosses (X) indicate intron-containing genes. Introns are distributed across 11 specific isotypes: Arg, Gln, Ile, Leu, Lys, Phe, Pro, Ser, Trp, Tyr, and Val. Notably, introns are absent from both the elongator (Met) and initiator (iMet) methionine tRNA families as well as in the Ala, Asn, Asp, Cys, Glu, Gly, His, and Thr isotypes in *S. schoenii*. Notably, introns are obligate in families such as Tyr-GTA and Pro-TGG, where every identified gene copy retains an intron.

53 nuclear tRNA genes contain introns, indicated by cross markers in Fig. 4 (*S. cerevisiae* has 61 as reported in Yoshihisa et al. 2007). These introns are not randomly distributed but are restricted to 11 specific isotypes: Arg, Gln, Ile, Leu, Lys, Phe, Pro, Ser, Trp, Tyr, and Val. Notably, introns appear to be obligate in families such as Tyr-GTA and Pro-TGG, where every gene copy retains an intron. This distribution is strongly consistent with *S. cerevisiae*, where introns are maintained in specific anticodon families for proper anticodon loop formation and modification (Pan 2018).

The mitochondrial genome encodes a distinct set of 20 tRNAs, with 15 predicted to be functional and 5 classified as pseudogenes (ChrM, Supplementary File 3). Unlike the nuclear repertoire, all mitochondrial tRNAs (mt-tRNAs) loci lack introns, consistent with Hao (2022), which shows that fungal mt-tRNA gene sets are usually intron-poor and the introns are instead concentrated in a subset of protein-coding and rRNA genes. Each mt-tRNA gene is present once in the mitochondrial contig, and its annotation is supported by Infernal (Inf) scores of ∼34 to 54 bits for most loci. However, 5 of these loci (decoding His, Lys, Ile, and 2 copies of Asp) were classified as pseudogenes due to low primary sequence conservation and an Inf score of <40 bits (Chan et al. 2021). The apparent loss of functional mt-tRNAs for these essential amino acids suggests that *S. schoenii* likely relies on the import of the corresponding cytosolic tRNAs to support mitochondrial translation. This compensatory mechanism of nuclear-encoded tRNAs into the mitochondria is well-documented in other fungal lineages where nuclear-encoded tRNAs are translocated across the mitochondrial membrane to rescue organellar defects (Schneider 2011).

#### LTR retrotransposons

**Fig. 5.**
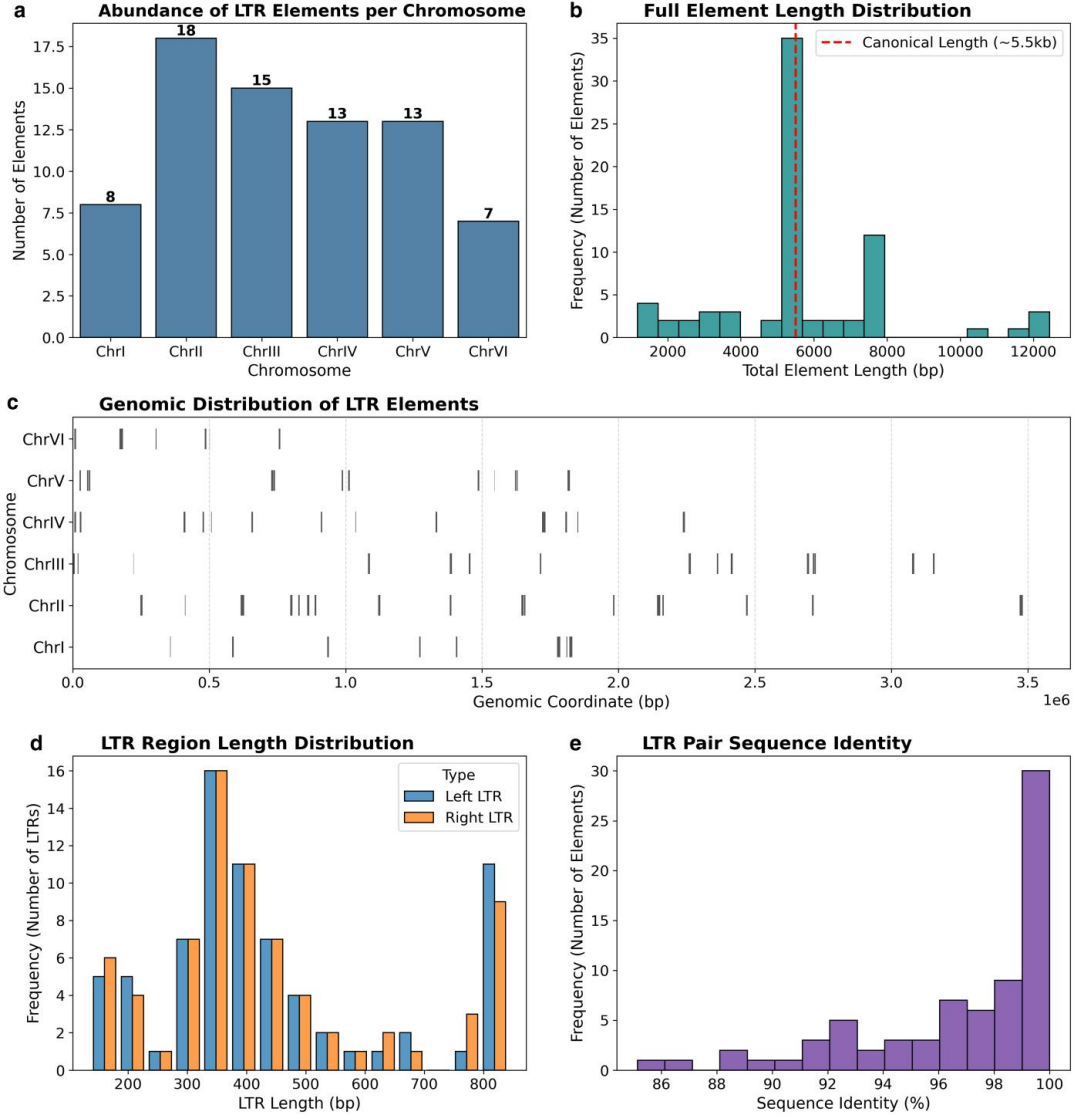
Characterization of the LTR retrotransposon landscape in *S. schoenii.* Panel a: Chromosomal abundance of the 74 identified full-length LTR retrotransposons. Elements are non-randomly distributed, with significant enrichment on chromosomes II and III. b) Size distribution of full-length elements. The prominent peak at ∼5.5 kb (dashed line) aligns with the canonical length of autonomous fungal retrotransposons (*e.g. Ty1*/Copia, *Ty3*/Gypsy), confirming the presence of intact, potentially active elements. c) Linear genomic map illustrating the physical distribution of retrotransposons across the 6 nuclear chromosomes. d) Length distribution of the individual LTR regions (left and right). The majority of LTRs fall within the typical 200 to 400 bp range required for promoter and terminator function. Panel e: Sequence identity between the paired 5′ and 3′ LTRs of each element. Since LTRs are identical upon insertion and diverge over time, the high identity (median 98.3%) indicates a recent burst of transpositional activity.

TEs, particularly LTR retrotransposons, are drivers of genomic plasticity in fungi, influencing genome size, gene expression, and chromosomal architecture (Bleykasten-Grosshans and Neuvéglise 2011; Muszewska et al. 2011). Structural signatures of retrotransposition were identified within the assembly, yielding 74 putative full-length LTR retrotransposons (Fig. 5a; Supplementary File 4). These elements are non-randomly distributed, with chromosomes II and III exhibiting the highest abundance (18 and 15 elements, respectively), while chromosome I harbors only 8 (Fig. 5a). As each element typically consists of 2 flanking LTRs, these 74 signatures represent approximately 148 distinct LTR sequences (Fig. 5d; Supplementary File 4).

Classification via protein domain profiling against the GyDB (Supplementary File 5) assigned 55 of the 74 elements (74%) into 2 major superfamilies based on conserved domain architecture: (a) *Ty1*/Copia (*Pseudoviridae*): The dominant superfamily in *S. schoenii*, comprising 47 elements. Lineage analysis shows that the majority belong to the “Pseudovirus” clade (27 elements), which is structurally homologous to the *Ty1* elements of *S. cerevisiae* (Qi et al. 2012). A subset of 15 elements was classified as “mixed”, likely reflecting the sequence divergence of these elements in non-model yeasts relative to reference databases. (b) *Ty3*/Gypsy (*Metaviridae*): Comprising 8 elements, primarily belonging to the Cer2-3 lineage (7 elements). This lineage corresponds to the Chromovirus group, which often contains a chromodomain targeting integration into heterochromatin or centromeric regions (Gao et al. 2008).

The identified elements range in length from 1.2 kb to 12.4 kb. As shown in Fig. 5b, the majority of loci cluster around the canonical 5 to 6 kb length typical of autonomous fungal retrotransposons such as *Ty1/Ty3*. However, exceptionally large elements (>10 kb) were also identified, most notably the 12.4 kb locus on chromosome IV (1,719,571 to 1,732,013) visible in both the length distribution and genomic map (Fig. 5b and c). Although this element was classified as *Ty1/*Copia (*Pseudovirus*) based on its conserved Reverse Transcriptase and RNase H domains, its length is more than double the size of a canonical Copia element. This discrepancy suggests that this feature may represent a nested insertion, where a newer element is likely integrated into an existing retrotransposon, expanding the element's size while retaining the original flanking LTRs. This phenomenon has been observed in *S. cerevisiae* and *Schizosaccharomyces pombe* and has been proposed as a strategy to avoid disruption of essential genes (Gao et al. 2008; Bleykasten-Grosshans and Neuvéglise 2011).

Further domain analysis confirmed that the identified elements encode highly conserved functional motifs. High coverage matches (>80% sequence coverage) were detected for all 4 essential domains required for retrotransposition: RT, INT, RNase H, and AP. The presence of these intact catalytic cores, alongside the conservation of LTR structural dimensions (Fig. 5d), indicates that *S. schoenii* retains potentially active autonomous elements. Notably, the elements classified as “mixed” lineages (15 Copia, 1 Gypsy) exhibit mosaic protein domains matching multiple reference clades (*e.g. Pseudovirus*, *Hydra*, and *Retrofit*). This reflects the specific evolutionary divergence of *S. schoenii* retrotransposons from the canonical reference elements defined in the GyDB.

Evolutionary timing of individual transposition events was estimated by analyzing the sequence identity between paired 5′ and 3′ LTRs (Fig. 5e). Because LTRs are identical at the time of insertion and diverge over time due to neutral mutation, their sequence similarity serves as a molecular clock (Havecker et al. 2004). The *S. schoenii* LTRs display remarkably high sequence identity, with a median similarity of 98.3%, and 19 elements (25.7%) possessing 100% identical LTRs (Median: 99.25% for Copia, 99.17% for Gypsy). This abundance of high-identity LTRs suggests a recent burst of transpositional activity in the *S. schoenii* lineage, distinguishing it from genomes dominated by ancient, degenerated solo LTRs (Carr et al. 2012). Another significant finding is the association of LTR retrotransposons with 2 regional centromeres. Genomic overlap analysis revealed 2 full-length elements intersecting the heterochromatic centromere boundaries of chromosomes III and IV, including a *Ty1*/Copia element (chromosome III: 2,717,808 to 2,723,473) exhibiting 100% LTR sequence identity (Repetitive Context; Supplementary Table 2). The localization of these newer elements suggests that the expansive, AT-rich pericentromeric cores of *S. schoenii* function as heterochromatic sinks containing recent retrotransposon insertions, characteristic of centromere-associated retrotransposons in other fungal genomes (Scott and Sullivan 2014).

#### Centromere identification and architecture

Manual Hi-C contact map inspection identified 6 candidate centromere loci for the nuclear chromosomes (Supplementary Table 2). The presence of strong 3D interaction peaks at these specific coordinates provided the initial biological evidence that *S. schoenii* maintains classical Rabl conformation typical of yeast nuclear architecture. Further screening yielded zero occurrences of the CDEI-spacer-CDEIII motif across these regions, supporting a regional rather than classical point-centromere architecture.

Given that regional centromeres are often associated with AT-rich heterochromatic sequences, a sliding-window GC-content analysis was performed, scanning the assembly to extract the highest, non-overlapping AT-rich peaks per chromosome: ChrI (74.78%), ChrII (76.24%), ChrIII (76.64%), ChrIV (75.36%), ChrV (75.46%), and ChrVI (75.46%). Across the combined 182,120 bp of all 6 cores, the global GC level fell down to just 33.16%. This high Adenine and Thymine (AT) enrichment increases DNA flexibility. While typical DNA wraps around standard histone (H3) proteins to form nucleosomes and compact the genome, this flexible, AT-rich environment is optimized for specialized Cse4 centromeric nucleosomes (Verdaasdonk and Bloom 2011). Cse4 nucleosomes are the structures that connect centromeric DNA to the kinetochore, thereby linking chromosomes to the mitotic spindle during cell division.

Additionally, isolating the boundaries of these AT-peaks helped estimate the centromere core sizes: ChrI: 26,781 bp, ChrII: 34,016 bp, ChrIII: 31,850 bp, ChrIV: 33,021 bp, ChrV: 25,953 bp, and ChrVI: 30,499 bp (Supplementary Table 2). These large sizes conclusively rule out the possibility of a point centromere architecture in *S. schoenii.* While *S. cerevisiae* uses short (∼125 bp) sequence-specific point centromeres located in gene-dense regions (Clarke and Carbon 1980), the kb-scale expansion observed here aligns structurally with the regional centromeres seen in *C. albicans* (3 to 5 kb) (Sanyal et al. 2004) and *S. pombe* (35 to 110 kb) (Wood et al. 2002).

Next, evidence of past transposable element activity was uncovered to determine the evolutionary drivers of this expansion. Highly diverged, incomplete LTR retrotransposons from the Copia superfamily were detected on 3 chromosomes. Decayed elements retaining AP (E-values 4.6e-11 and 2.2e-10), INT, RT, and RNaseH domains were found on ChrIII and ChrI, whereas ChrVI contained a heavily fragmented element retaining only AP and INT domains. RepeatMasker also flagged an 82 bp *Ty1*/Copia fragment. Because all detected elements are incomplete, this pattern indicates that retrotransposons may have previously occupied these regions and subsequently decayed, suggesting that current centromere organization likely relies on other repetitive structures.

Subsequent TRF analysis revealed that while satellite DNA arrays are present in a chromosome-specific manner, they only account for about 1.24% of the total pericentromeric sequence. Taken together with the RepeatMasker findings, the data indicates that the bulk of these massive 25 to 34 kb AT-rich centromeric cores consists of complex, non-tandem sequence interspersed with small satellite islands (147 bp repeat unit on ChrI with a very high TRF alignment score of 500, arrays built from 87, 109, and 110 bp repeat units on ChrV, 61 bp repeats on ChrIII/ChrVI, and shorter 22 and 32 bp units on ChrII and ChrIV, respectively). Taken together, the structural LTR and TRF analyses point to a complex architecture for the *S. schoenii* centromeres. The regions are defined by expansive, non-tandem AT-rich cores that also serve as sinks for retrotransposons. Alongside the highly decayed Copia remnants, genomic overlap analysis identified 2 intact, full-length LTR elements overlapping the centromere boundaries on ChrIII and ChrIV (Supplementary Table 2). The organization of this structure, featuring a central satellite array flanked by retrotransposon insertions overlapping these regions, resembles the pericentromeric heterochromatin found in *S. pombe* (Wood et al. 2002).

**Fig. 6.**
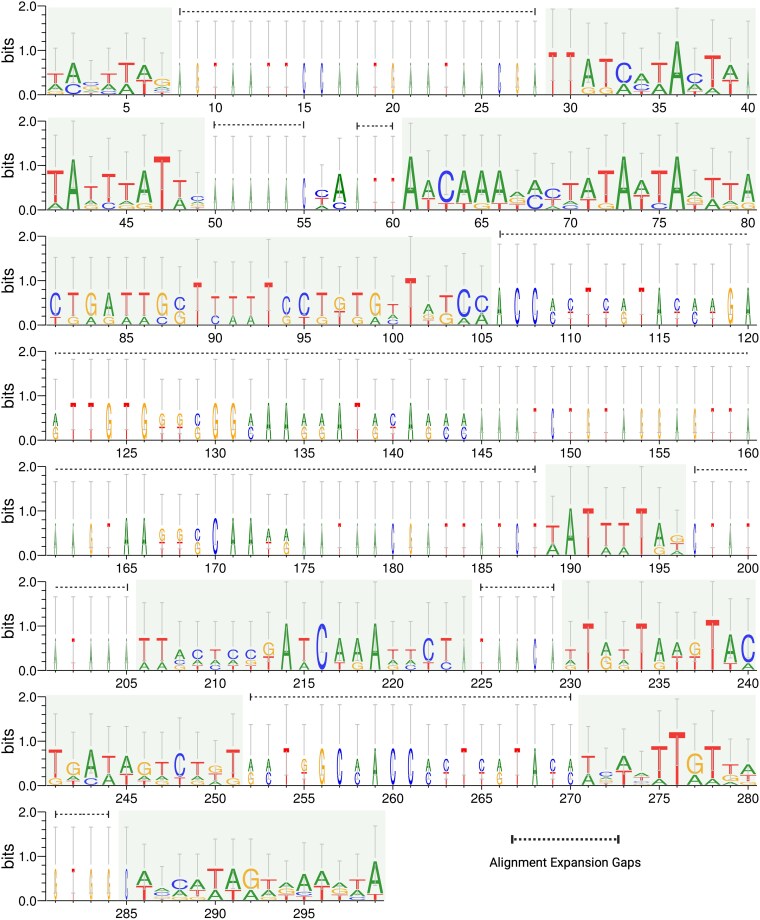
Conserved architecture of chromosome-specific centromeric satellite DNA in *S schoenii.* Satellite consensus sequences from all 6 chromosomes (32 to 147 bp) were aligned using MUSCLE and visualized as a sequence logo. The *y*-axis (Shannon entropy, measured in bits) quantifies nucleotide conservation at each alignment position, with a maximum value of 2.0 bits indicating absolute sequence identity across all 6 chromosomes. Shaded boxes highlight distinct, highly conserved AT-tracts. The *x*-axis (299 positions) and the corresponding unshaded, low-conservation regions reflect insertion/deletion gaps generated during the alignment of the varying-length periodic units. The conservation of these AT-dominant (or GC depleted) regions across all 6 chromosomes, despite the differing lengths of the underlying satellite arrays, identifies them as the essential domains for kinetochore (CENP-A/Cse4) recruitment.

To evaluate conserved sequence features across all 6 centromeric loci, the dominant repeat consensus sequences (from the TRF analysis) were structurally aligned and visualized as a sequence logo (Fig. 6). The resulting sequence logo shows the evolutionary dynamics of these centromeres: while the overall length of the repeating units varies between chromosomes (represented by the alignment gaps along the extended *x*-axis in Fig. 6), the overlapping DNA sequences are strongly conserved. These highly conserved, AT-rich regions (green blocks in Fig. 6) with high information content (and low Shannon entropy) serve as the rigid AT-anchors required for kinetochore attachment to the chromosome (Allshire and Karpen 2008; Plohl et al. 2014). Additionally, visualizing the physical distribution of these arrays within the full chromosomal context confirms this architecture. In every instance, the globally offset satellite arrays physically align within the big, gene-poor intergenic gaps. This verifies that these 25 to 34 kb AT-rich, repeat-dense regions are strictly located between conserved housekeeping genes (Supplementary File 2).

Using the defined centromere boundaries and total chromosome lengths, the morphological type of each chromosome was classified based on the physical long-arm to short-arm (q/p) ratio. The *S. schoenii* genome consists primarily of acrocentric and submetacentric chromosomes. Chromosomes I and VI are submetacentric (ratios of 2.36 and 2.94, respectively), while chromosomes II, III, and IV are distinctly acrocentric (ratios ranging from 3.67 to 6.16). Notably, chromosome V exhibits a strictly telocentric architecture (ratio of 98.66), with the centromere positioned a mere ∼22.5 kb from the extreme right telomeric boundary.

#### Mating-type switching

**Fig. 7.**
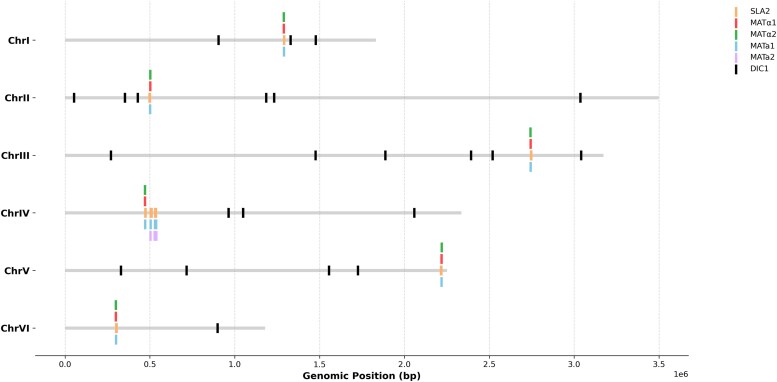
Genomic distribution map of *MAT* loci and flanking markers across the 6 nuclear chromosomes of *S. schoenii.* The chromosomal map displays the genomic coordinates of mating-type genes (*MATa1, MATa2, MATα1, MATα2*) and the ancestral flanking genes (*SLA2 and DIC1*). Horizontal gray bars represent the individual chromosomes, with colored vertical lines indicating the location of each specific gene hit. The map illustrates the genome-wide expansion and dispersion of the *SLA2-MAT* cassettes. While the *SLA2* anchor gene (orange) maintains tight physical linkage with the dispersed *MAT* orthologs on every chromosome, the *DIC1* gene (black) is widely scattered throughout the genome, demonstrating a complete spatial dissociation from the ancestral *SLA2-MAT-DIC1* synteny. Notably, Chromosome IV harbors three distinct *SLA2-MAT* casettes.

To understand the genetic basis of homothallism in *S. schoenii*, the genomic architecture of the *MAT* loci was investigated. In *S. cerevisiae*, the active *MAT* locus is a contiguous sequence flanked by the conserved anchor genes *SLA2* and *DIC1* (Haber 2012; Krassowski et al. 2019). Although this *SLA2-MAT-DIC1* arrangement represents the ancestral state of budding yeasts, the *MAT* locus is known to be evolutionarily dynamic (Gordon et al. 2011; Krassowski et al. 2019; Wolfe and Butler 2022). In fact, homology searches of *C. albicans* and *S. cerevisiae* orthologs against our *S. schoenii* genome revealed an interesting reorganization of this ancestral synteny. While *SLA2* remains closely linked to the *MAT* orthologs, *DIC1* is spatially dissociated from the *MAT* clusters (Fig. 7, Supplementary Table 4). More importantly, the analysis also shows a substantial genomic expansion of *MAT* cassettes. Unlike the compact ∼10 kb system (1 active locus, 2 silent heterochromatic reservoirs) of *S. cerevisiae* (Haber 2012; Krassowski et al. 2019), *S. schoenii* contains *SLA2-MAT* cassettes across all 6 chromosomes, occupying a ∼42.8 kb genomic footprint (Fig. 7; Supplementary Tables 4 and 6). While unusual in budding yeasts, analogous expansions occur in basidiomycetes like *Cryptococcus neoformans*, where suppressed recombination captures adjacent genes and repetitive elements to generate a >100 kb *MAT* locus (Fraser et al. 2004; Sun et al. 2019).

To determine if and how the structural organization of these loci translates into functional transcripts, RNA-seq splice junctions were cross-referenced against the genomic boundaries of all *MAT* and *SLA2* gene orthologs, and interestingly, they showed evidence of co-transcription and intergenic splicing across the expanded loci (Supplementary Table 5). Across all *MAT* cassettes, splice junctions (189 to 247 bp in length) were identified, which perfectly correspond to the intergenic boundaries between the *MATa1* and *SLA2* coding sequences (also visible in IGV snapshots in Supplementary File 6). Additionally, conserved 58 bp introns excise the *MATa2-MATa1* intergenic space within the central and right *MAT* sub-clusters on chromosome IV. Previous studies have demonstrated that yeast RNA Polymerase II can read through terminators, creating large, overlapping multi-gene transcripts (Pelechano et al. 2013). As the spliceosome strictly operates *in cis* upon a single pre-mRNA transcript, the precise excision of sequencing reads physically bridging the 3′ terminus of *MATa1* and the 5′ start of *SLA2* in our data supports that this intergenic spacer is functioning as an intron. The targeted excision of this spacer strongly suggests that *MATa1* and *SLA2* are co-transcribed to produce *MATa1-SLA2* chimeric transcripts. While further molecular validation is required to determine whether these chimeric transcripts are translated into protein, the presence of conserved intergenic splicing suggests that *S. schoenii* maintains tight linkage of the *SLA2-MATa* region to support continuous co-transcription.

Mapping these cassettes against the regional centromeres revealed that this expanded MAT system is not randomly dispersed across euchromatic arms but is tethered to pericentromeric heterochromatin (Supplementary Table 6; Supplementary Fig. 3). While the left and right *MAT* sub-clusters on chromosome IV are euchromatic (residing ∼23 and 20 kb away from the heterochromatin, respectively), the remainder of the expanded system is closer to the centromeres. The *SLA2-MAT* loci on chromosomes II, III, and V are situated within 3.2 to 15.8 kb of the heterochromatic boundaries, using these spatial gaps as structural buffers to maintain transcription (Supplementary Table 6; Supplementary Fig. 3). Conversely, the *MAT* cassettes on chromosomes I, the central cluster of chromosome IV, and chromosome VI overlap with the centromeric boundaries (Supplementary Fig. 3). As expected for genes embedded in centromeric heterochromatin, these *MAT* genes show reduced RNA-seq coverage compared with their fully euchromatic paralogs, but they are not completely silenced. Because the splicing analysis suggests co-transcription with the *SLA2* anchor, this basal *MAT* expression is likely driven by transcription of the *SLA2* gene. Additionally, the IGV visualization shows that even when overlapping with the centromere, the read depth of the *SLA2* anchor remains higher than that of the adjacent *MAT* genes, consistent with a highly expressed housekeeping gene vs lower-expressed transcription factors. These observations suggest that *S. schoenii* may have active euchromatic islands within its heterochromatin. Another evidence for it is the complete absence of tRNAs within the centromere-embedded *MAT* cassettes on chromosomes I, IV, and VI which suggests that *S. schoenii* lacks the usual tRNA-based barrier elements at these loci. Instead, the sustained RNA polymerase II-driven transcription of the adjacent *SLA2* gene likely maintains a local euchromatic island and acts as the primary barrier preventing full heterochromatic silencing of the *MAT* genes. This is consistent with the works of Donze and Kamakaka (2001) and Wang et al. (2014), who show that highly expressed RNA Polymerase II promoters are known to actively work against repressive chromatin. Further translated homology searches of unannotated, highly transcribed peaks in this region show presence of a conserved repeat element (homologous to the *S. cerevisiae* YJR098C) tightly flanking these embedded *MAT* cassettes. It also resolved internal transcriptional gaps within the cassettes themselves while uncovering highly diverged *SLA2* anchor genes on chromosomes IV (472,227 to 475,000 bp) and VI (300,500 to 302,471 bp). Confirming the presence of these genes is consistent with the *SLA2-MAT* euchromatic islands remaining structurally intact and transcriptionally active. Finally, positioning the MAT genes around the repressive centromere potentially provides the protected environment expected for homothallic switching mechanisms, while also preventing these genes from shuffling during meiosis to help the resulting spores remain compatible for self-fertilization (Menkis et al. 2008; Branco et al. 2017; Carpentier et al. 2019).

**Fig. 8.**
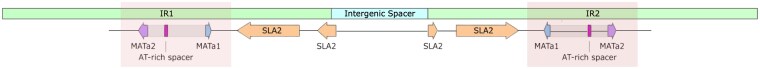
Map of the palindromic mating-type sub-cluster on chromosome IV in *S. schoenii.* Physical map detailing the genomic architecture of the palindromic cassette (527,000 to 539,000 bp) of the mating-type locus in Chromosome IV of *S. schoenii*. Block arrows denote the position, length, and transcriptional orientation of the *MATa1, MATa2*, and *SLA2* ORFs). The locus is characterized by a central *SLA2* anchor gene (orange) and it is flanked upstream by an inverted *MATa1* (blue) and forward *MATa2* (purple), and downstream by a mirrored *MATa1* and *MATa2* array. AT-rich intergenic spacer regions (magenta), representing bidirectional promoters, are located between the divergent *MAT* sequences. This palindromic cassette also consists of 2 large, inverted repeats (IR1, IR2) flanking an intergenic spacer.

While the centromere governs much of this architecture, further structural analysis shows that the euchromatic arms of chromosome IV serve as the primary location for active MAT expression. Spanning a 67,168 bp region (470,610 to 537,777 bp) interrupted by the centromeric core, this locus is partitioned into distinct functional sub-clusters. The leftmost sub-cluster (470,610 to 475,000 bp) serves as an active ɑ-dominant region, transcribing *MATɑ2* and *MATɑ1*, while the rightmost sub-cluster (528,044 to 537,777 bp) forms a highly expressed palindromic cassette. Sequence alignment shows *MATa2* (←) and *MATa1* (←) genes flanking the left side of the *SLA2* anchor, mirrored by direct repeat *MATa1* (→) and *MATa2* (→) genes on the right side (Fig. 8). Within this palindrome, an AT-rich intergenic spacer region was identified between the divergent *MATa2* and *MATa1* genes. In previously characterized homothallic yeasts that utilize inversion switching, similar AT-rich spacers function as bidirectional promoters facilitating the physical flipping of the chromosomal segment (Hanson et al. 2014; Wolfe and Butler 2022). RNA-seq data confirms that the conserved *MATa1*-*MATa2* intergenic splicing junction is perfectly mirrored within this palindromic array (junctions at 529,128 bp and 536,635 bp). The high expression of both *MATa* and *MATɑ* euchromatic regions across chromosome IV, paired with this complex intergenic splicing, is consistent with an unsynchronized, homothallic population actively undergoing MAT switching (Haber 2012; Riley et al. 2016).

Numerous macro-palindromes (arm lengths >100 bp) are distributed across multiple chromosomes in *S. schoenii* (Supplementary Table 7). The frequency and scale of these structures suggest the genome has a natural tendency to form large, inverted repeats. Interestingly, 3 of the *MAT* cassettes are specifically encapsulated by this palindromic architecture. This includes the *MAT* cassette on chromosome III, which is situated within a 7.2 kb spacer flanked by 124 bp inverted repeats, and the *MAT* cluster on chromosome V that is encapsulated within a 7.4 kb spacer bounded by 139 bp inverted repeats. But the most prominent example is the active *MAT* locus on chromosome IV. While this chromosome contains 3 distinct macro-palindromes, 1 specifically encapsulates the right *MAT* cassette (*MATa*-dominant). This massive 19.1 kb structure consists of two 8.5 kb inverted repeats or palindromic arms (523,326 to 531,900 bp and 533,920 to 542,495 bp) that share 99.98% sequence identity and flank a 2,019 bp spacer (Fig. 8). The right *MAT* cassette is physically embedded mostly within the palindromic arms themselves. This unique architecture suggests a functional inversion mechanism driven by the reorientation of the spacer, likely containing a promoter or other regulatory element for this *MAT* cassette. Because the palindromic arms provide extensive homology, the chromosome may undergo intrachromosomal homologous recombination, potentially inverting the spacer, thereby reorienting its internal regulatory elements to move away from the *SLA2-MATa* cluster in 1 palindromic arm and align them instead with the *SLA2-MATa* cluster in the opposite arm (similar to a transcriptional toggle). Finally, the structural encapsulation of these specific *MAT* cassettes across multiple independent loci suggests that *S. schoenii* may be employing large-scale chromosomal inversions to regulate its MAT locus, which likely also facilitated the mobility and duplication of the *SLA2-MAT* region.

### Comparative genomics and gene family evolution

**Fig. 9.**
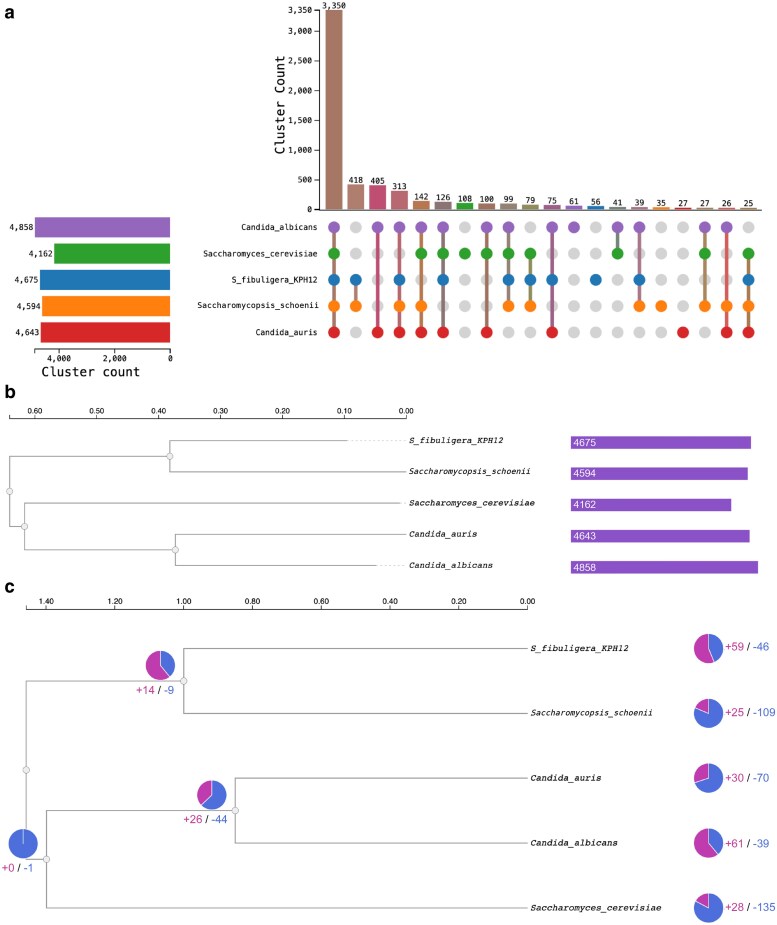
Comparative genomic analysis of *S. schoenii.* Panel a: Distribution of 5,685 orthologous clusters across 5 yeast species (*S. schoenii*, *S. fibuligera*, *S. cerevisiae*, *C. albicans*, *C. auris*). The largest intersection (3,350 clusters) represents core orthogroups conserved across all species. Species-specific orthogroups are also shown. Horizontal bars indicate total cluster counts per species. Only the top 20 orthologous cluster combinations, ranked by cluster count, are shown. Panel b: Phylogenetic tree inferred from orthologous clusters using Maximum Likelihood. Branch lengths represent evolutionary distance (scale bar: 0.1 substitutions per site). *S. schoenii* and *S. fibuligera* form a monophyletic clade within *Saccharomycopsis*, with *S. cerevisiae* as an outgroup to the *Candida* clade. Colored bars indicate total orthogroup counts per species. Panel c: Gene family expansion (magenta) and contraction (blue) along the species tree. *S. schoenii* experienced 25 expansions and 109 contractions post-divergence from *S. fibuligera*. The *Saccharomycopsis* ancestral lineage shows 14 expansions and 9 contractions. Numbers at nodes and tips show expansion and contraction event counts.

Comparative genomic analysis with 4 related yeasts, the fermentative *S. fibuligera* (KPH12), the model yeast *S. cerevisiae*, and the pathogenic *C. albicans* and *C. auris*, clarified the placement of *S. schoenii* within Saccharomycotina and highlighted gene family patterns associated with its necrotrophic lifestyle (Fig. 9). Across the 5 species, 5,685 orthologous clusters were identified, of which 3,350 (59%) were core orthogroups conserved in all species, representing shared housekeeping and fundamental cellular functions (Fig. 9a). *S. schoenii* and *S. fibuligera* shared 418 clusters (7.4%; Fig. 9a) uniquely, likely reflecting genus-level adaptations, including traits related to their specialized ecology and, in *S. schoenii*, predation. An additional 313 clusters (5.5%; Fig. 9a) were shared by *S. schoenii*, *S. fibuligera*, and the 2 *Candida* species but absent from *S. cerevisiae*, a pattern consistent with CTG clade-specific features such as the reassignment of the CTG codon to serine (Santos et al. 2011).

Maximum Likelihood phylogeny supports the fact that *S. schoenii* and *S. fibuligera* are sister species, forming a monophyletic *Saccharomycopsis* clade (Fig. 9b). This clade diverged from the common ancestor of the *Saccharomyces* and *Candida* lineages at a branch length of ∼0.4 substitutions per site, consistent with substantial evolutionary separation while retaining conserved Saccharomycetales features. The 2 *Candida* species formed a distinct pathogenic clade, with *S. cerevisiae* positioned as an outgroup, matching established yeast phylogenies and validating the orthology framework used for gene family analysis (Gabaldón et al. 2016).

Gene family evolution revealed a pronounced asymmetry in gains and losses in *S. schoenii* after divergence from *S. fibuligera*. A total of 25 gene family expansions and 109 contractions were detected (Fig. 9c), indicating genomic remodeling in *S. schoenii*, associated with its specialization for predation. The largest expansion, cluster2 (54 genes; GO:0006508; *P* = 7.2 × 10^−67^), encodes aspartic-type endopeptidases (secreted aspartic proteases), key enzymes in degrading fungal cell walls and proteins and thus directly relevant to necrotrophic attack (Junker et al. 2019). Additional expansions were enriched for genes implicated in biofilm formation and adhesion (cluster30, 16 genes; GO:0090606), cell wall degrading enzymes associated with penetration peg formation (cluster139, 10 genes; GO:0000920), and extracellular secreted proteins (cluster401, 7 genes; GO:0005576). Parallel expansions in transmembrane transporters (clusters 49, 207; GO:0055085) and specialized nutrient transporters (cluster784, 6 genes; GO:0042906, xanthine transport) suggest enhanced uptake of prey-derived metabolites, while expansions in oxidative stress response genes (cluster237, 8 genes; GO:0034599) likely support management of reactive oxygen species generated during the metabolic demands of predation.

Conversely, the 109 contracted gene families suggest a loss of functions less critical for a predatory lifestyle. The major contraction involved transposon-related genes (cluster1, 57 genes; GO:0015074; *P* = 1.8 × 10^−49^), indicating a reduction in mobile genetic elements and a trend toward genomic stabilization, reminiscent of patterns observed in certain obligate parasites (Keeling and Slamovits 2004). Contractions in protein O-linked glycosylation genes (clusters 10, 25; 54 genes; GO:0006493; *P* = 2.4 × 10^−26^) align with differences in cell wall composition relative to model budding yeasts and could reflect a cell surface optimized for prey interaction rather than host immune evasion (Aimanianda et al. 2009). Reductions in gene families involved in drug/xenobiotic transport (cluster75, 11 genes; GO:0006855; *P* = 5.9 × 10^−19^) and amino acid transmembrane transport (cluster13, 20 genes; GO:0003333; *P* = 5.0 × 10^−13^) may suggest decreased reliance on general nutrient uptake mechanisms, consistent with a predatory lifestyle where nutrients are obtained primarily from prey digestion (Junker et al. 2019).

These patterns of *S. schoenii* differ from those seen in more generalist pathogens such as *C. albicans*, which retain an extensive repertoire of genes for stress tolerance, host interaction, and metabolic flexibility (Butler et al. 2009; Gabaldón et al. 2016). In *S. schoenii*, the bias toward contraction (109 lost vs 25 gained families), coupled with focused expansions in proteases, adhesins, transporters, and oxidative stress genes, points to a genome that has been tuned for predatory efficiency.

### Summary

The chromosome-scale assembly of *Saccharomycopsis schoenii* provides a highly contiguous 14.3 Mb reference genome resolved into 6 nuclear and 1 mitochondrial chromosome. This evidence-annotated resource details the species’ compact gene structure, regional centromere organization, an expanded and co-transcribed MAT architecture, and specific gene family adaptations associated with its necrotrophic lifestyle. It may serve as a resource for future comparative studies investigating the molecular basis of fungal predation and yeast evolution.

## Supplementary Material

jkag067_Supplementary_Data

## Data Availability

The *Saccharomycopsis schoenii* genome assembly is publicly available in NCBI GenBank under the accession JBTFFQ000000000. The version described in this paper is version JBTFFQ010000000. The BioProject accession number is PRJNA1364152 and the raw sequencing data for PacBio, Hi-C, and RNA-seq are deposited in the NCBI Sequence Read Archive (SRA) and are available via https://www.ncbi.nlm.nih.gov/sra/PRJNA1364152. The gene annotation file (GTF) generated in this study is available as Supplementary File 1. The custom Python and shell scripts utilized for the analyses described in this manuscript are available on GitHub (https://github.com/dkriti/Saccharomycopsis-schoenii-Genome-Assembly) and have been archived on Zenodo (https://doi.org/10.5281/zenodo.18950794). Supplemental material available at G3 online.

## References

[jkag067-B1] Aimanianda V et al 2009. Surface hydrophobin prevents immune recognition of airborne fungal spores. Nature. 460:1117–1121. 10.1038/nature08264. 19713928

[jkag067-B2] Allshire RC, Karpen GH. 2008. Epigenetic regulation of centromeric chromatin: old dogs, new tricks? Nat Rev Genet. 9:923–937. 10.1038/nrg2466. 19002142 PMC2586333

[jkag067-B3] Andrews S . 2010. FastQC: a quality control tool for high throughput sequence data. http://www.bioinformatics.babraham.ac.uk/projects/fastqc/.

[jkag067-B4] Ashburner M et al 2000. Gene ontology: tool for the unification of biology. Nat Genet. 25:25–29. 10.1038/75556. 10802651 PMC3037419

[jkag067-B5] Benson G . 1999. Tandem repeats finder: a program to analyze DNA sequences. Nucleic Acids Res. 27:573–580. 10.1093/nar/27.2.573. 9862982 PMC148217

[jkag067-B6] Bleykasten-Grosshans C, Neuvéglise C. 2011. Transposable elements in yeasts. C R Biol. 334:679–686. 10.1016/j.crvi.2011.05.017. 21819950

[jkag067-B7] Bloom-Ackermann Z et al 2014. A comprehensive tRNA deletion library unravels the genetic architecture of the tRNA pool. PLoS Genet. 10:e1004084. 10.1371/journal.pgen.1004084. 24453985 PMC3894157

[jkag067-B8] Bon E et al 2003. Molecular evolution of eukaryotic genomes: hemiascomycetous yeast spliceosomal introns. Nucleic Acids Res. 31:1121–1135. 10.1093/nar/gkg213. 12582231 PMC150231

[jkag067-B9] Branco S et al 2017. Evolutionary strata on young mating-type chromosomes despite the lack of sexual antagonism. Proc Natl Acad Sci U S A. 114:7067–7072. 10.1073/pnas.1701658114. 28630332 PMC5502610

[jkag067-B10] Brůna T, Hoff KJ, Lomsadze A, Stanke M, Borodovsky M. 2021. BRAKER2: automatic eukaryotic genome annotation with GeneMark-EP+ and AUGUSTUS supported by a protein database. NAR Genom Bioinform. 3:lqaa108. 10.1093/nargab/lqaa108. 33575650 PMC7787252

[jkag067-B11] Brůna T, Lomsadze A, Borodovsky M. 2020. GeneMark-EP+: eukaryotic gene prediction with self-training in the space of genes and proteins. NAR Genom Bioinform. 2:lqaa026. 10.1093/nargab/lqaa026. 32440658 PMC7222226

[jkag067-B12] Butler G et al 2009. Evolution of pathogenicity and sexual reproduction in eight *Candida* genomes. Nature. 459:657–662. 10.1038/nature08064. 19465905 PMC2834264

[jkag067-B13] Camacho C et al 2009. BLAST+: architecture and applications. BMC Bioinformatics. 10:421. 10.1186/1471-2105-10-421. 20003500 PMC2803857

[jkag067-B14] Cantalapiedra CP, Hernández-Plaza A, Letunic I, Bork P, Huerta-Cepas J. 2021. eggNOG-mapper v2: functional annotation, orthology assignments, and domain prediction at the metagenomic scale. Mol Biol Evol. 38:5825–5829. 10.1093/molbev/msab293. 34597405 PMC8662613

[jkag067-B15] Carpentier F et al 2019. Convergent recombination cessation between mating-type genes and centromeres in selfing anther-smut fungi. Genome Res. 29:944–953. 10.1101/gr.242578.118. 31043437 PMC6581054

[jkag067-B16] Carr M, Bensasson D, Bergman CM. 2012. Evolutionary genomics of transposable elements in *Saccharomyces cerevisiae*. PLoS One. 7:e50978. 10.1371/journal.pone.0050978. 23226439 PMC3511429

[jkag067-B17] Chan PP, Lin BY, Mak AJ, Lowe TM. 2021. tRNAscan-SE 2.0: improved detection and functional classification of transfer RNA genes. Nucleic Acids Res. 49:9077–9096. 10.1093/nar/gkab688. 34417604 PMC8450103

[jkag067-B18] Chan PP, Lowe TM. 2016. GtRNAdb 2.0: an expanded database of transfer RNA genes identified in complete and draft genomes. Nucleic Acids Res. 44:D184–D189. 10.1093/nar/gkv1309. 26673694 PMC4702915

[jkag067-B19] Choo JH et al 2016. Whole-genome *de novo* sequencing, combined with RNA-Seq analysis, reveals unique genome and physiological features of the amylolytic yeast *Saccharomycopsis fibuligera* and its interspecies hybrid. Biotechnol Biofuels. 9:246. 10.1186/s13068-016-0653-4. 27872659 PMC5106798

[jkag067-B20] Clarke L, Carbon J. 1980. Isolation of a yeast centromere and construction of functional small circular chromosomes. Nature. 287:504–509. 10.1038/287504a0. 6999364

[jkag067-B21] Crooks GE, Hon G, Chandonia J-M, Brenner SE. 2004. WebLogo: a sequence logo generator. Genome Res. 14:1188–1190. 10.1101/gr.849004. 15173120 PMC419797

[jkag067-B22] d’Enfert C . 2004. CandidaDB: a genome database for *Candida albicans* pathogenomics. Nucleic Acids Res. 33:D353–D357. 10.1093/nar/gki124. PMC540078 15608215

[jkag067-B23] Dobin A et al 2013. STAR: ultrafast universal RNA-seq aligner. Bioinformatics. 29:15–21. 10.1093/bioinformatics/bts635. 23104886 PMC3530905

[jkag067-B24] Donze D, Kamakaka RT. 2001. RNA polymerase III and RNA polymerase II promoter complexes are heterochromatin barriers in *Saccharomyces cerevisiae*. EMBO J. 20:520–531. 10.1093/emboj/20.3.520. 11157758 PMC133458

[jkag067-B25] Duan Z et al 2010. A three-dimensional model of the yeast genome. Nature. 465:363–367. 10.1038/nature08973. 20436457 PMC2874121

[jkag067-B26] Dudchenko O et al 2018. The juicebox assembly tools module facilitates de novo assembly of mammalian genomes with chromosome-length scaffolds for under $1000 [preprint]. bioRxiv 254797. 10.1101/254797.

[jkag067-B27] Dujon B et al 2004. Genome evolution in yeasts. Nature. 430:35–44. 10.1038/nature02579. 15229592

[jkag067-B28] Durand NC et al 2016. Juicebox provides a visualization system for Hi-C contact maps with unlimited zoom. Cell Syst. 3:99–101. 10.1016/j.cels.2015.07.012. 27467250 PMC5596920

[jkag067-B29] Edgar RC . 2004. MUSCLE: multiple sequence alignment with high accuracy and high throughput. Nucleic Acids Res. 32:1792–1797. 10.1093/nar/gkh340. 15034147 PMC390337

[jkag067-B30] Ellinghaus D, Kurtz S, Willhoeft U. 2008. LTRharvest, an efficient and flexible software for de novo detection of LTR retrotransposons. BMC Bioinformatics. 9:18. 10.1186/1471-2105-9-18. 18194517 PMC2253517

[jkag067-B31] Emms DM, Kelly S. 2019. OrthoFinder: phylogenetic orthology inference for comparative genomics. Genome Biol. 20:238. 10.1186/s13059-019-1832-y. 31727128 PMC6857279

[jkag067-B32] Fatema U, Broberg A, Jensen DF, Karlsson M, Dubey M. 2018. Functional analysis of polyketide synthase genes in the biocontrol fungus *Clonostachys rosea*. Sci Rep. 8:15009. 10.1038/s41598-018-33391-1. 30301915 PMC6177402

[jkag067-B33] Fraser JA et al 2004. Convergent evolution of chromosomal sex-determining regions in the animal and fungal kingdoms. PLoS Biol. 2:e384. 10.1371/journal.pbio.0020384. 15538538 PMC526376

[jkag067-B34] Gabaldón T, Naranjo-Ortíz MA, Marcet-Houben M. 2016. Evolutionary genomics of yeast pathogens in the Saccharomycotina. FEMS Yeast Res. 16:fow064. 10.1093/femsyr/fow064. 27493146 PMC5815160

[jkag067-B35] Gao X, Hou Y, Ebina H, Levin HL, Voytas DF. 2008. Chromodomains direct integration of retrotransposons to heterochromatin. Genome Res. 18:359–369. 10.1101/gr.7146408. 18256242 PMC2259100

[jkag067-B36] Goffeau A et al 1996. Life with 6000 genes. Science. 274:546–567. 10.1126/science.274.5287.546. 8849441

[jkag067-B37] Gordon JL et al 2011. Evolutionary erosion of yeast sex chromosomes by mating-type switching accidents. Proc Natl Acad Sci U S A. 108:20024–20029. 10.1073/pnas.1112808108. 22123960 PMC3250169

[jkag067-B38] Gremme G, Steinbiss S, Kurtz S. 2013. GenomeTools: a comprehensive software library for efficient processing of structured genome annotations. IEEE/ACM Trans Comput Biol and Bioinf. 10:645–656. 10.1109/TCBB.2013.68. 24091398

[jkag067-B39] Groenewald M et al 2023. A genome-informed higher rank classification of the biotechnologically important fungal subphylum *Saccharomycotina*. Stud Mycol. 105:1–22. 10.3114/sim.2023.105.01. 38895705 PMC11182611

[jkag067-B40] Gurevich A, Saveliev V, Vyahhi N, Tesler G. 2013. QUAST: quality assessment tool for genome assemblies. Bioinformatics. 29:1072–1075. 10.1093/bioinformatics/btt086. 23422339 PMC3624806

[jkag067-B41] Haber JE . 2012. Mating-type genes and *MAT* switching in *Saccharomyces cerevisiae*. Genetics. 191:33–64. 10.1534/genetics.111.134577. 22555442 PMC3338269

[jkag067-B42] Hajihosseinali M, Nasr S, Amoozegar MA, Yurkov A. 2020. *Saccharomycopsis oxydans* sp. nov., a new non-fermentative member in the genus Saccharomycopsis isolated from a traditional dairy product of Iran. Int J Syst Evol Microbiol. 70:1059–1063. 10.1099/ijsem.0.003874. 31746728

[jkag067-B43] Hanson SJ, Byrne KP, Wolfe KH. 2014. Mating-type switching by chromosomal inversion in methylotrophic yeasts suggests an origin for the three-locus *Saccharomyces cerevisiae* system. Proc Natl Acad Sci U S A. 111:E4851–E4858. 10.1073/pnas.1416014111. 25349420 PMC4234585

[jkag067-B44] Hao W . 2022. From genome variation to molecular mechanisms: what we have learned from yeast mitochondrial genomes? Front Microbiol. 13:806575. 10.3389/fmicb.2022.806575. 35126340 PMC8811140

[jkag067-B45] Havecker ER, Gao X, Voytas DF. 2004. The diversity of LTR retrotransposons. Genome Biol. 5:225. 10.1186/gb-2004-5-6-225. 15186483 PMC463057

[jkag067-B46] Hesselbart A, Junker K, Wendland J. 2018. Draft genome sequence of *Saccharomycopsis fermentans* CBS 7830, a predacious yeast belonging to the Saccharomycetales. Genome Announc. 6:e01445-17. 10.1128/genomeA.01445-17. 29326220 PMC5764944

[jkag067-B47] Hooks KB, Delneri D, Griffiths-Jones S. 2014. Intron evolution in Saccharomycetaceae. Genome Biol Evol. 6:2543–2556. 10.1093/gbe/evu196. 25364803 PMC4202332

[jkag067-B48] Hoyer LL . 2023. Extraction of yeast high-molecular-weight genomic DNA. Protocols.io. 10.17504/protocols.io.rm7vzb1b4vx1/v1.

[jkag067-B49] Janbon G, et al 2014. Analysis of the genome and transcriptome of *Cryptococcus neoformans* var. *grubii* reveals complex RNA expression and microevolution leading to virulence attenuation. PLoS Genet. 10:e1004261. 10.1371/journal.pgen.1004261. 24743168 PMC3990503

[jkag067-B50] Jones DT, Taylor WR, Thornton JM. 1992. The rapid generation of mutation data matrices from protein sequences. Bioinformatics. 8:275–282. 10.1093/bioinformatics/8.3.275. 1633570

[jkag067-B51] Junker K et al 2018. The mycoparasitic yeast *Saccharomycopsis schoenii* predates and kills multi-drug resistant *Candida auris*. Sci Rep. 8:14959. 10.1038/s41598-018-33199-z. 30297756 PMC6175896

[jkag067-B52] Junker K, Chailyan A, Hesselbart A, Forster J, Wendland J. 2019. Multi-omics characterization of the necrotrophic mycoparasite *Saccharomycopsis schoenii*. PLoS Pathog. 15:e1007692. 10.1371/journal.ppat.1007692. 31071195 PMC6508603

[jkag067-B53] Kanehisa M, Furumichi M, Sato Y, Kawashima M, Ishiguro-Watanabe M. 2023. KEGG for taxonomy-based analysis of pathways and genomes. Nucleic Acids Res. 51:D587–D592. 10.1093/nar/gkac963. 36300620 PMC9825424

[jkag067-B54] Karlsson M et al 2015. Insights on the evolution of mycoparasitism from the genome of *Clonostachys rosea*. Genome Biol Evol. 7:465–480. 10.1093/gbe/evu292. 25575496 PMC4350171

[jkag067-B55] Karlsson M, Atanasova L, Jensen DF, Zeilinger S. 2017. Necrotrophic mycoparasites and their genomes. Microbiol Spectr. 5:5.2.08. 10.1128/microbiolspec.FUNK-0016-2016. PMC11687461 28281442

[jkag067-B56] Keeling PJ, Slamovits CH. 2004. Simplicity and complexity of microsporidian genomes. Eukaryot Cell. 3:1363–1369. 10.1128/EC.3.6.1363-1369.2004. 15590811 PMC539024

[jkag067-B57] Krassowski T et al 2018. Evolutionary instability of CUG-Leu in the genetic code of budding yeasts. Nat Commun. 9:1887. 10.1038/s41467-018-04374-7. 29760453 PMC5951914

[jkag067-B58] Krassowski T et al 2019. Multiple reinventions of mating-type switching during budding yeast evolution. Curr Biol. 29:2555–2562.e8. 10.1016/j.cub.2019.06.056. 31353182 PMC6692504

[jkag067-B59] Lachance M-A, Pang W-M. 1997. Predacious yeasts. Yeast. 13:225–232. 10.1002/(SICI)1097-0061(19970315)13:3<225::AID-YEA87>3.0.CO;2-I. 9090051

[jkag067-B60] Levan A, Fredga K, Sandberg AA. 1964. Nomenclature for centromeric position on chromosomes. Hereditas. 52:201–220. 10.1111/j.1601-5223.1964.tb01953.x.

[jkag067-B61] Li H et al 2009. The sequence alignment/map format and SAMtools. Bioinformatics. 25:2078–2079. 10.1093/bioinformatics/btp352. 19505943 PMC2723002

[jkag067-B62] Li H . 2013. Aligning sequence reads, clone sequences and assembly contigs with BWA-MEM [preprint]. arXiv, arXiv:1303.3997. 10.48550/ARXIV.1303.3997.

[jkag067-B63] Li H. 2020. auN: a new metric to measure assembly contiguity. auN: a new metric to measure assembly contiguity. https://lh3.github.io/2020/04/08/a-new-metric-on-assembly-contiguity.

[jkag067-B64] Lieberman-Aiden E et al 2009. Comprehensive mapping of long-range interactions reveals folding principles of the human genome. Science. 326:289–293. 10.1126/science.1181369. 19815776 PMC2858594

[jkag067-B65] Llorens C et al 2011. The gypsy database (GyDB) of mobile genetic elements: release 2.0. Nucleic Acids Res. 39:D70–D74. 10.1093/nar/gkq1061. 21036865 PMC3013669

[jkag067-B66] Manni M, Berkeley MR, Seppey M, Simão FA, Zdobnov EM. 2021. BUSCO update: novel and streamlined workflows along with broader and deeper phylogenetic coverage for scoring of eukaryotic, prokaryotic, and viral genomes. Mol Biol Evol. 38:4647–4654. 10.1093/molbev/msab199. 34320186 PMC8476166

[jkag067-B67] Mendes FK, Vanderpool D, Fulton B, Hahn MW. 2021. CAFE 5 models variation in evolutionary rates among gene families. Bioinformatics. 36:5516–5518. 10.1093/bioinformatics/btaa1022. 33325502

[jkag067-B68] Menkis A, Jacobson DJ, Gustafsson T, Johannesson H. 2008. The mating-type chromosome in the filamentous ascomycete Neurospora tetrasperma represents a model for early evolution of sex chromosomes. PLoS Genet. 4:e1000030. 10.1371/journal.pgen.1000030. 18369449 PMC2268244

[jkag067-B69] Mitrovich QM, Tuch BB, Guthrie C, Johnson AD. 2007. Computational and experimental approaches double the number of known introns in the pathogenic yeast *Candida albicans*. Genome Res. 17:492–502. 10.1101/gr.6111907. 17351132 PMC1832096

[jkag067-B70] Muszewska A, Hoffman-Sommer M, Grynberg M. 2011. LTR retrotransposons in fungi. PLoS One. 6:e29425. 10.1371/journal.pone.0029425. 22242120 PMC3248453

[jkag067-B71] Neuvéglise C, Marck C, Gaillardin C. 2011. The intronome of budding yeasts. C R Biol. 334:662–670. 10.1016/j.crvi.2011.05.015. 21819948

[jkag067-B72] Ó Cinnéide E, Scaife C, Dillon ET, Wolfe KH. 2024. Evolution of the genetic code in the Ascoideales (CUG-Ser2) yeast clade: the ancestral tRNA-Leu(CAG) gene is retained in most *Saccharomycopsis* Species but is nonessential and not used for translation. Genome Biol Evol. 16:evae166. 10.1093/gbe/evae166. 39081261 PMC11342251

[jkag067-B73] Pan T . 2018. Modifications and functional genomics of human transfer RNA. Cell Res. 28:395–404. 10.1038/s41422-018-0013-y. 29463900 PMC5939049

[jkag067-B74] Parenteau J et al 2019. Introns are mediators of cell response to starvation. Nature. 565:612–617. 10.1038/s41586-018-0859-7. 30651641

[jkag067-B75] Pelechano V, Wei W, Steinmetz LM. 2013. Extensive transcriptional heterogeneity revealed by isoform profiling. Nature. 497:127–131. 10.1038/nature12121. 23615609 PMC3705217

[jkag067-B76] Pimenta RS et al 2008. Biological control of *Penicillium italicum, P. digitatum* and *P. expansum* by the predacious yeast *Saccharomycopsis schoenii* on oranges. Braz J Microbiol. 39:85–90. 10.1590/S1517-838220080001000020. 24031185 PMC3768350

[jkag067-B77] Plohl M, Meštrović N, Mravinac B. 2014. Centromere identity from the DNA point of view. Chromosoma. 123:313–325. 10.1007/s00412-014-0462-0. 24763964 PMC4107277

[jkag067-B78] Qi X et al 2012. Retrotransposon profiling of RNA polymerase III initiation sites. Genome Res. 22:681–692. 10.1101/gr.131219.111. 22287102 PMC3317150

[jkag067-B79] Quinlan AR, Hall IM. 2010. BEDTools: a flexible suite of utilities for comparing genomic features. Bioinformatics. 26:841–842. 10.1093/bioinformatics/btq033. 20110278 PMC2832824

[jkag067-B80] Rabl C . 1885. Über Zelltheilung. Morphologisches Jahrbuch. 10:214–330. https://scholar.google.com/scholar_lookup?journal=Morphologisches%20Jahrbuch&title=%C3%9Cber%20zelltheilung&author=C%20Rabl&volume=10&publication_year=1885&pages=214-330&#d=gs_cit&t=1774332086799&u=%2Fscholar%3Fq%3Dinfo%3Abkj98br500oJ%3Ascholar.google.com%2F%26output%3Dcite%26scirp%3D0%26hl%3Den.

[jkag067-B81] Riley R et al 2016. Comparative genomics of biotechnologically important yeasts. Proc Natl Acad Sci U S A. 113:9882–9887. 10.1073/pnas.1603941113. 27535936 PMC5024638

[jkag067-B82] Saha S, Bridges S, Magbanua ZV, Peterson DG. 2008. Computational approaches and tools used in identification of dispersed repetitive DNA sequences. Tropical Plant Biol. 1:85–96. 10.1007/s12042-007-9007-5.

[jkag067-B83] Santos MAS, Gomes AC, Santos MC, Carreto LC, Moura GR. 2011. The genetic code of the fungal CTG clade. C R Biol. 334:607–611. 10.1016/j.crvi.2011.05.008. 21819941

[jkag067-B84] Sanyal K, Baum M, Carbon J. 2004. Centromeric DNA sequences in the pathogenic yeast *Candida albicans* are all different and unique. Proc Natl Acad Sci U S A. 101:11374–11379. 10.1073/pnas.0404318101. 15272074 PMC509209

[jkag067-B85] Schneider A . 2011. Mitochondrial tRNA import and its consequences for mitochondrial translation. Annu Rev Biochem. 80:1033–1053. 10.1146/annurev-biochem-060109-092838. 21417719

[jkag067-B86] Scott KC, Sullivan BA. 2014. Neocentromeres: a place for everything and everything in its place. Trends Genet. 30:66–74. 10.1016/j.tig.2013.11.003. 24342629 PMC3913482

[jkag067-B87] Stajich JE, Dietrich FS, Roy SW. 2007. Comparative genomic analysis of fungal genomes reveals intron-rich ancestors. Genome Biol. 8:R223. 10.1186/gb-2007-8-10-r223. 17949488 PMC2246297

[jkag067-B88] Stanke M, Diekhans M, Baertsch R, Haussler D. 2008. Using native and syntenically mapped cDNA alignments to improve *de novo* gene finding. Bioinformatics. 24:637–644. 10.1093/bioinformatics/btn013. 18218656

[jkag067-B89] Storer J, Hubley R, Rosen J, Wheeler TJ, Smit AF. 2021. The Dfam community resource of transposable element families, sequence models, and genome annotations. Mob DNA. 12:2. 10.1186/s13100-020-00230-y. 33436076 PMC7805219

[jkag067-B90] Subramanian A et al 2005. Gene set enrichment analysis: a knowledge-based approach for interpreting genome-wide expression profiles. Proc Natl Acad Sci U S A. 102:15545–15550. 10.1073/pnas.0506580102. 16199517 PMC1239896

[jkag067-B91] Sun J et al 2023. OrthoVenn3: an integrated platform for exploring and visualizing orthologous data across genomes. Nucleic Acids Res. 51:W397–W403. 10.1093/nar/gkad313. 37114999 PMC10320085

[jkag067-B92] Sun S, Coelho MA, Heitman J, Nowrousian M. 2019. Convergent evolution of linked mating-type loci in basidiomycete fungi. Giraud T, editor. PLoS Genet. 15:e1008365. 10.1371/journal.pgen.1008365. 31490920 PMC6730849

[jkag067-B93] Tegenfeldt F et al 2025. OrthoDB and BUSCO update: annotation of orthologs with wider sampling of genomes. Nucleic Acids Res. 53:D516–D522. 10.1093/nar/gkae987. 39535043 PMC11701741

[jkag067-B94] The Gene Ontology Consortium et al 2026. The gene ontology knowledgebase in 2026. Nucleic Acids Res. 54:D1779–D1792. 10.1093/nar/gkaf1292. 41413728 PMC12807639

[jkag067-B95] Thorvaldsdóttir H, Robinson JT, Mesirov JP. 2013. Integrative genomics viewer (IGV): high-performance genomics data visualization and exploration. Brief Bioinform. 14:178–192. 10.1093/bib/bbs017. 22517427 PMC3603213

[jkag067-B96] Verdaasdonk JS, Bloom K. 2011. Centromeres: unique chromatin structures that drive chromosome segregation. Nat Rev Mol Cell Biol. 12:320–332. 10.1038/nrm3107. 21508988 PMC3288958

[jkag067-B97] Wang J, Lawry ST, Cohen AL, Jia S. 2014. Chromosome boundary elements and regulation of heterochromatin spreading. Cell Mol Life Sci. 71:4841–4852. 10.1007/s00018-014-1725-x. 25192661 PMC4234687

[jkag067-B98] Warburton PE, Giordano J, Cheung F, Gelfand Y, Benson G. 2004. Inverted repeat structure of the human genome: the X-chromosome contains a preponderance of large, highly homologous inverted repeats that contain testes genes. Genome Res. 14:1861–1869. 10.1101/gr.2542904. 15466286 PMC524409

[jkag067-B99] Wolfe KH, Butler G. 2022. Mating-type switching in budding yeasts, from flip/flop inversion to cassette mechanisms. Microbiol Mol Biol Rev. 86:e00007-21. 10.1128/mmbr.00007-21. 35195440 PMC8941940

[jkag067-B100] Wolff J et al 2020. Galaxy HiCExplorer 3: a web server for reproducible Hi-C, capture Hi-C and single-cell Hi-C data analysis, quality control and visualization. Nucleic Acids Res. 48:W177–W184. 10.1093/nar/gkaa220. 32301980 PMC7319437

[jkag067-B101] Wood V et al 2002. The genome sequence of *Schizosaccharomyces pombe*. Nature. 415:871–880. 10.1038/nature724. 11859360

[jkag067-B102] Yoshihisa T, Ohshima C, Yunoki-Esaki K, Endo T. 2007. Cytoplasmic splicing of tRNA in *Saccharomyces cerevisiae*. Genes Cells. 12:285–297. 10.1111/j.1365-2443.2007.01056.x. 17352735

[jkag067-B103] Yuan X et al 2021. Complete genomic characterization and identification of *Saccharomycopsisphalluae* sp. nov., a novel pathogen causes yellow rot disease on Phallus rubrovolvatus. J Fungi (Basel). 7:707. 10.3390/jof7090707. 34575745 PMC8468998

[jkag067-B104] Zhang R-G et al 2022. TEsorter: an accurate and fast method to classify LTR-retrotransposons in plant genomes. Hortic Res. 9:uhac017. 10.1093/hr/uhac017. 35184178 PMC9002660

[jkag067-B105] Zhou C, McCarthy SA, Durbin R. 2023. YaHS: yet another Hi-C scaffolding tool. Bioinformatics. 39:btac808. 10.1093/bioinformatics/btac808. 36525368 PMC9848053

