# Supplementary material for: Chromosome-scale genome assembly and characterization of Saccharomycopsis schoenii, a necrotrophic predatory yeast
Source: G3 (Bethesda). 2026 Mar 18;16(5):jkag067. doi: 10.1093/g3journal/jkag067 (PMC13148404; doi:10.1093/g3journal/jkag067)
Supplement: jkag067_Supplementary_Data [file jkag067_supplementary_data.zip › Supplemental_Material_legends_G3-2026-406693.docx]

## **Supporting information**

### **Supplementary Figure 1. High molecular weight DNA extraction**

This is an electropherogram of high molecular weight (HMW) DNA extracted from *S. schoenii*. The profile shows a dominant peak corresponding to ~60 kb fragments, indicating successful recovery of long DNA suitable for PacBio sequencing. The electropherogram was generated on Tapestation (Agilent, 5067-5366 and 5067-5365).

**Supplementary Figure 2. Distribution of intron lengths in the *S. schoenii* genome**

The frequency histogram displays the length distribution of all 884 annotated introns. The distribution is positively skewed, with a median length of 113 bp (red dashed line) and a mean length of 259.8 bp (blue dashed line). The long tail is driven by conserved genes with long, regulatory introns that are >700 bp (*e.g.*, *DBP2, YRA1*), while the peak corresponds to the canonical short introns found in the majority of genes (*e.g.*, *VMA* subunits).

**Supplementary Figure 3. Snapgene visualizations of Mating-Type (*MAT*) cassettes across the *S. schoenii* nuclear genome**

The snapgene visualizations of the eight *MAT* cassettes across the *S. schoenii* nuclear genome, shows the conservation of the *SLA2* anchor gene across all loci along with their spatial relationship to the regional centromeres. Chromosomes II, III, and V maintain pericentromeric gaps ranging from 3.2 kb to 15.8 kb. Chromosome IV contains a left and a right euchromatic cassette located ~23 kb and ~20 kb away from the heterochromatic boundary. The *MAT* cassettes on Chromosomes I, VI, and the central cluster of Chromosome IV physically intersect the regional centromeres.

### **Supplementary Table 1. Hi‑C Library QC metrics for *S. schoenii* (XLSX)**

### **Supplementary Table 2. Centromere coordinates of nuclear chromosomes in *S. schoenii* (XLSX)**

### **Supplementary Table 3. Intron lengths in conserved and regulatory gene families (XLSX)**

### **Supplementary Table 4. BLAST Results for *MAT* loci (XLSX)**

### **Supplementary Table 5. Conserved Intergenic Splicing Across Expanded *MAT* Clusters (XLSX)**

### **Supplementary Table 6. Spatial and Functional Organization of *MAT* Loci in *S. schoenii* (XLSX)**

### **Supplementary Table 7. Genome-Wide Palindrome and *MAT* Locus Overlap Analysis** **(XLSX)**

### **Supplementary File 1. *S. schoenii* draft genome annotation (GTF)**

**Supplementary File 2: Genomic organization of *S. schoenii* regional centromeres (PDF)**

**Supplementary File 3. Annotated tRNA repertoire of *S. schoenii* (TXT)**

**Supplementary File 4. LTR retrotransposon landscape in *S. schoenii* (TXT)**

**Supplementary File 5. Evolutionary classification of Transposable elements in *S. schoenii* (TSV)**

**Supplementary File 6. *MAT* cassette + centromere coverage in RNA‑seq IGV snapshots (PDF)**
