# Supplementary material for: Chromosome-scale genome assembly and characterization of Saccharomycopsis schoenii, a necrotrophic predatory yeast
Source: G3 (Bethesda). 2026 Mar 18;16(5):jkag067. doi: 10.1093/g3journal/jkag067 (PMC13148404; doi:10.1093/g3journal/jkag067)
Supplement: jkag067_Supplementary_Data [file jkag067_supplementary_data.zip › Supplementary_File_2_(PDF)_G3-2026-406693.pdf]

IGV snapshots for centromere placement:

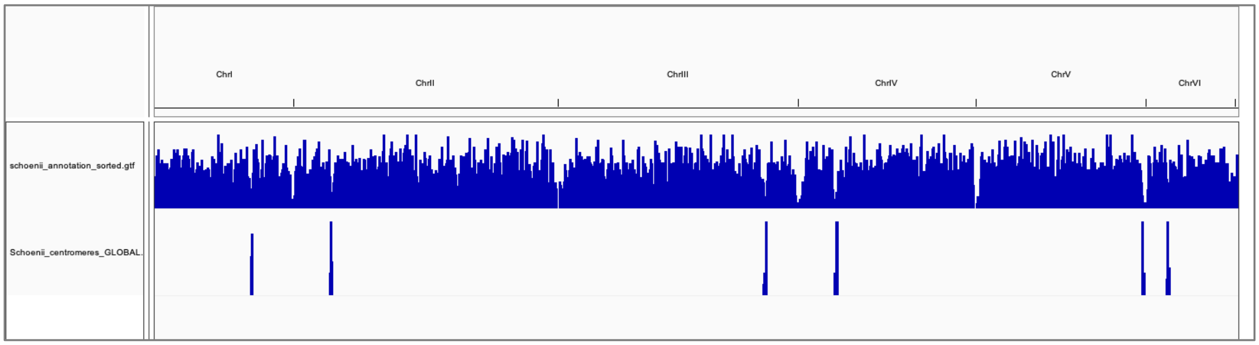

Hi-C contact map confirming the centromere placements:

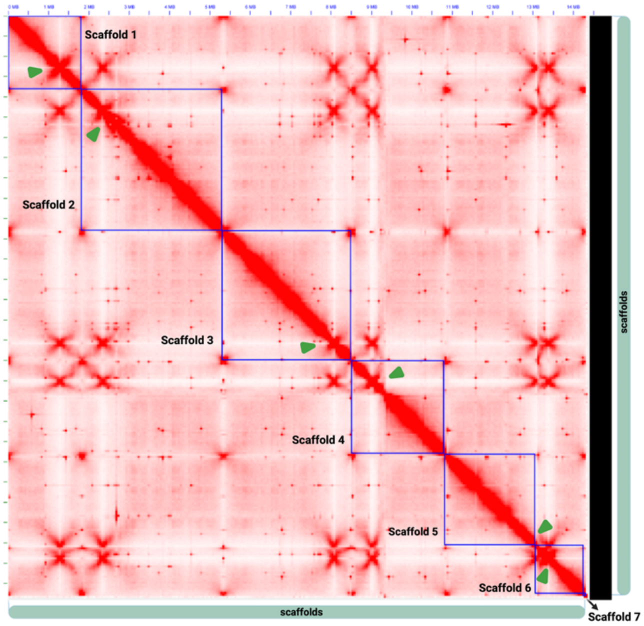

Chromosome-specific IGV snapshots (Genomic organization of *Saccharomycopsis schoenii* regional centromeres):

Integrative Genomics Viewer (IGV) visualization of the six nuclear chromosomes showing the mapped centromeric satellite arrays (bottom tracks) aligned against the whole-genome assembly and gene annotations (top blue tracks). The *de novo* identified satellite DNA localizes exclusively to large (25.9-34.0 kb), gene-depleted intergenic regions. Across all chromosomes, these repeat-dense pericentromeric boundaries are closely flanked by conserved housekeeping genes.

ChrI:

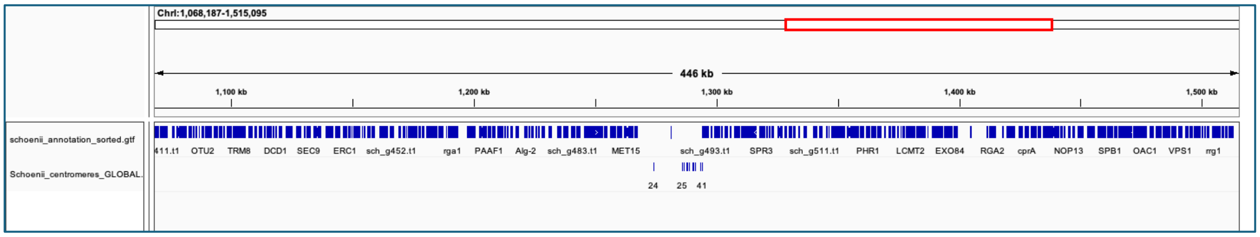

ChrII:

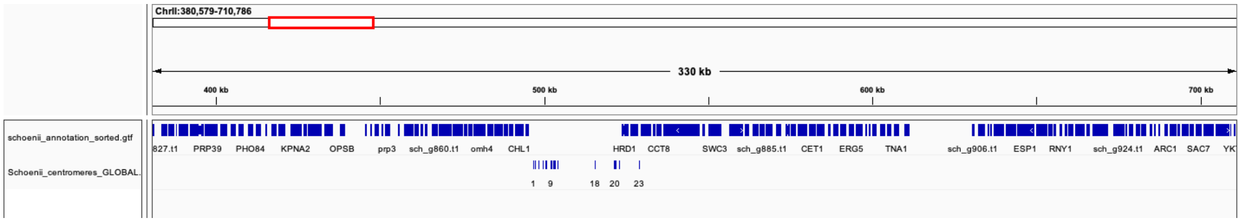

ChrIII:

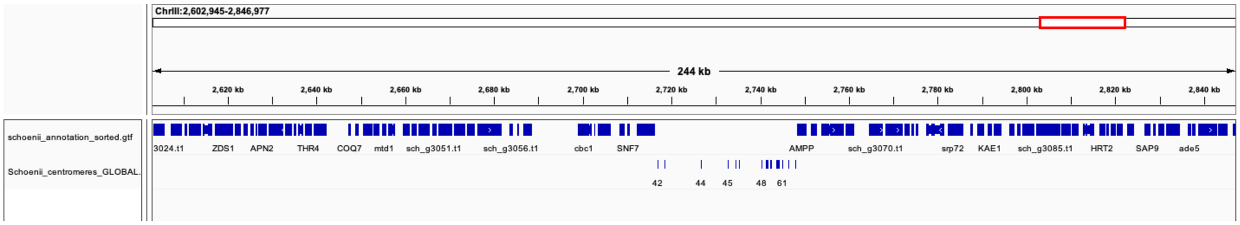

ChrIV:

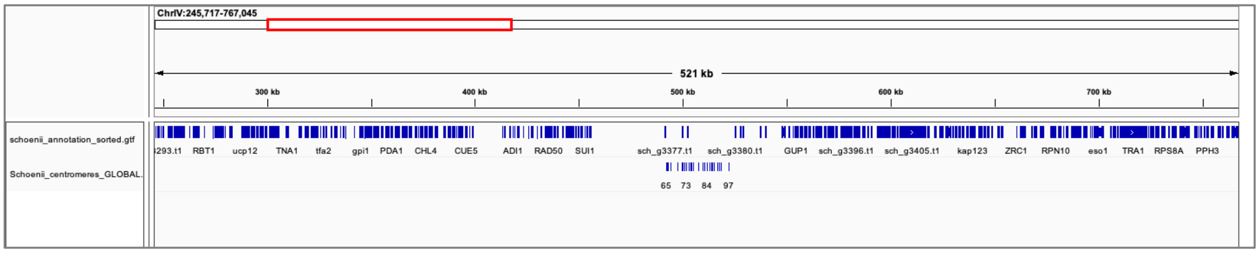

ChrV:

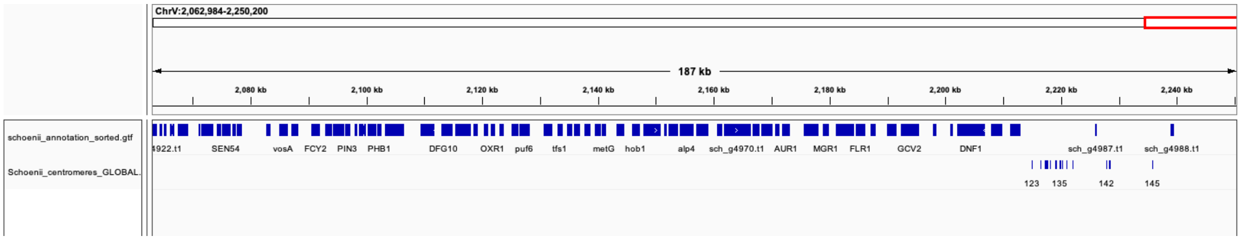
ChrV:2,062,984-2,250,200
187 kb
2,080 kb
2,100 kb
2,120 kb
2,140 kb
2,160 kb
2,180 kb
2,200 kb
2,220 kb
2,240 kb
schoenii_annotation_sorted.gtf
Schoenii_centromeres_GLOBAL.
SEN54
vosA
FCY2
PIN3
PHB1
DFG10
OXR1
puf6
tfs1
metG
hob1
alp4
sch_g4970.t1
AUR1
MGR1
FLR1
GCV2
DNF1
sch_g4987.t1
sch_g4988.t1
123
135
142
145

ChrVI

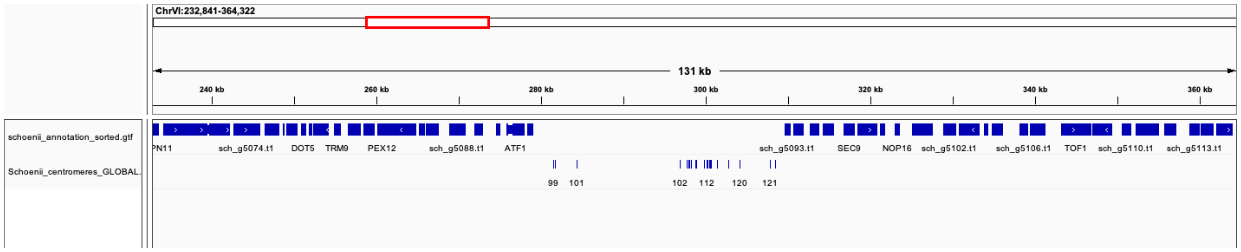
ChrVI:232,841-364,322
131 kb
240 kb
260 kb
280 kb
300 kb
320 kb
340 kb
360 kb
schoenii_annotation_sorted.gtf
Schoenii_centromeres_GLOBAL.
sch_g5074.t1
DOT5
TRM9
PEX12
sch_g5088.t1
ATF1
sch_g5093.t1
SEC9
NOP16
sch_g5102.t1
sch_g5106.t1
TOF1
sch_g5110.t1
sch_g5113.t1
99
101
102
112
120
121
