# Supplementary material for: Chromosome-scale genome assembly and characterization of Saccharomycopsis schoenii, a necrotrophic predatory yeast
Source: G3 (Bethesda). 2026 Mar 18;16(5):jkag067. doi: 10.1093/g3journal/jkag067 (PMC13148404; doi:10.1093/g3journal/jkag067)

ChrI:1,280,001-1,291,822 (MAT cassette + centromere):

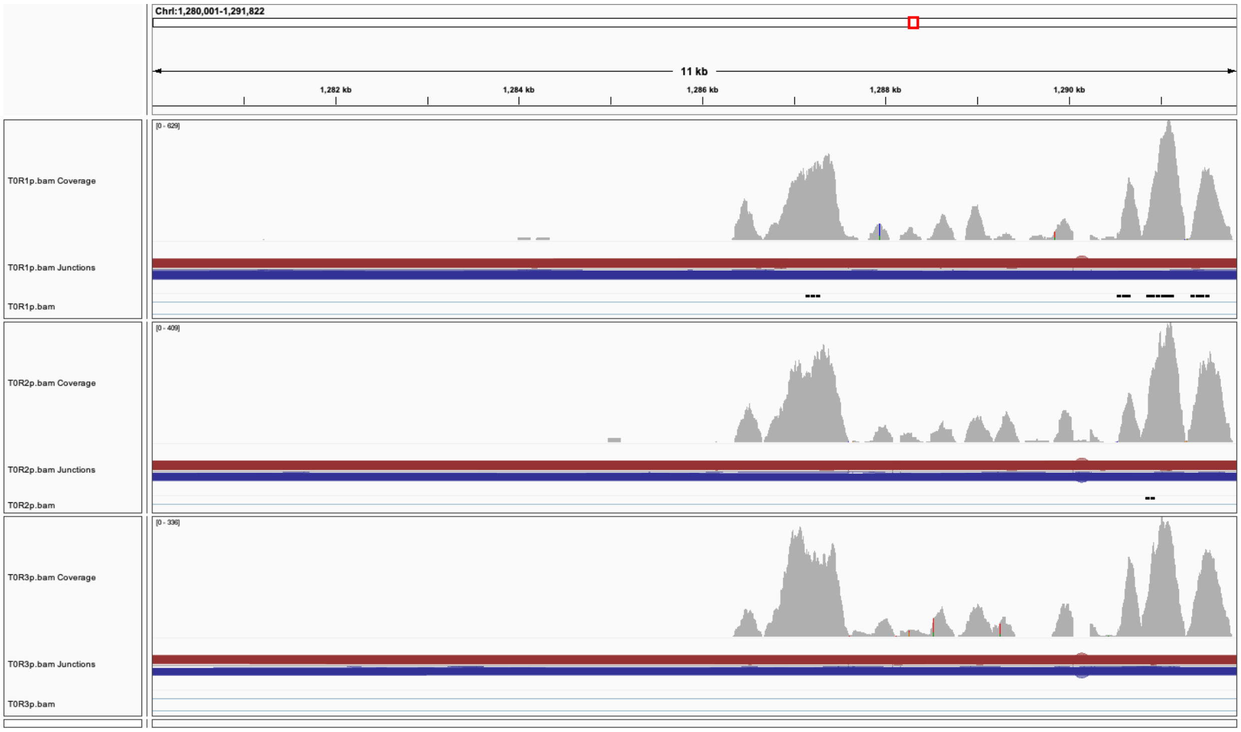

ChrII:497,890-528,405 (MAT cassette + centromere):

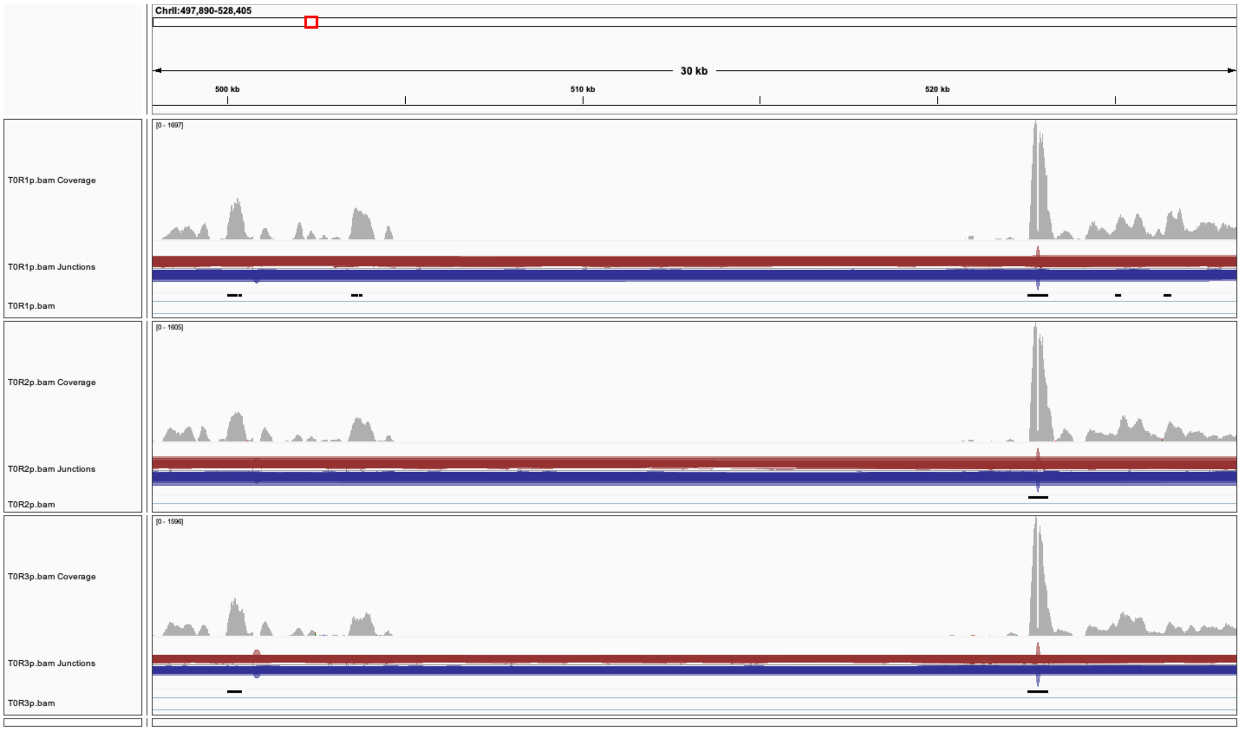

ChrIII:2,720,440-2,746,903 (MAT cassette + centromere):

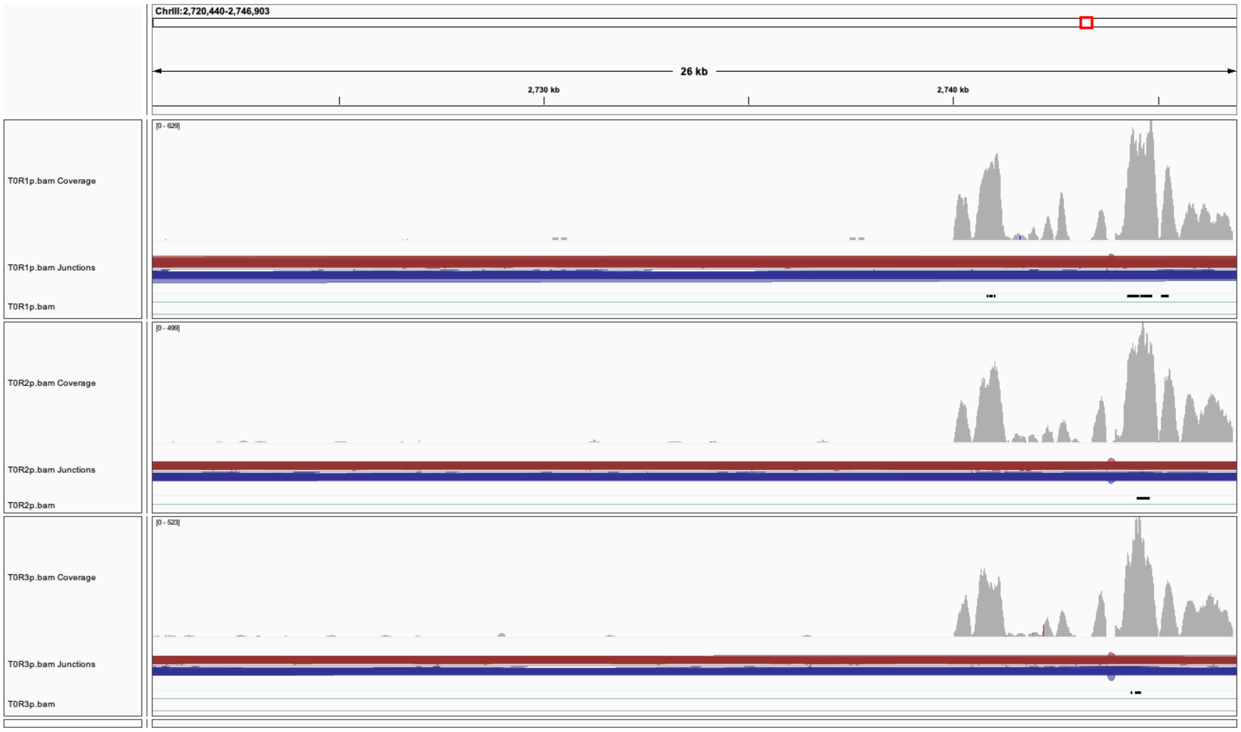

ChrIV:470,610-475,000 (Left MAT cassette):

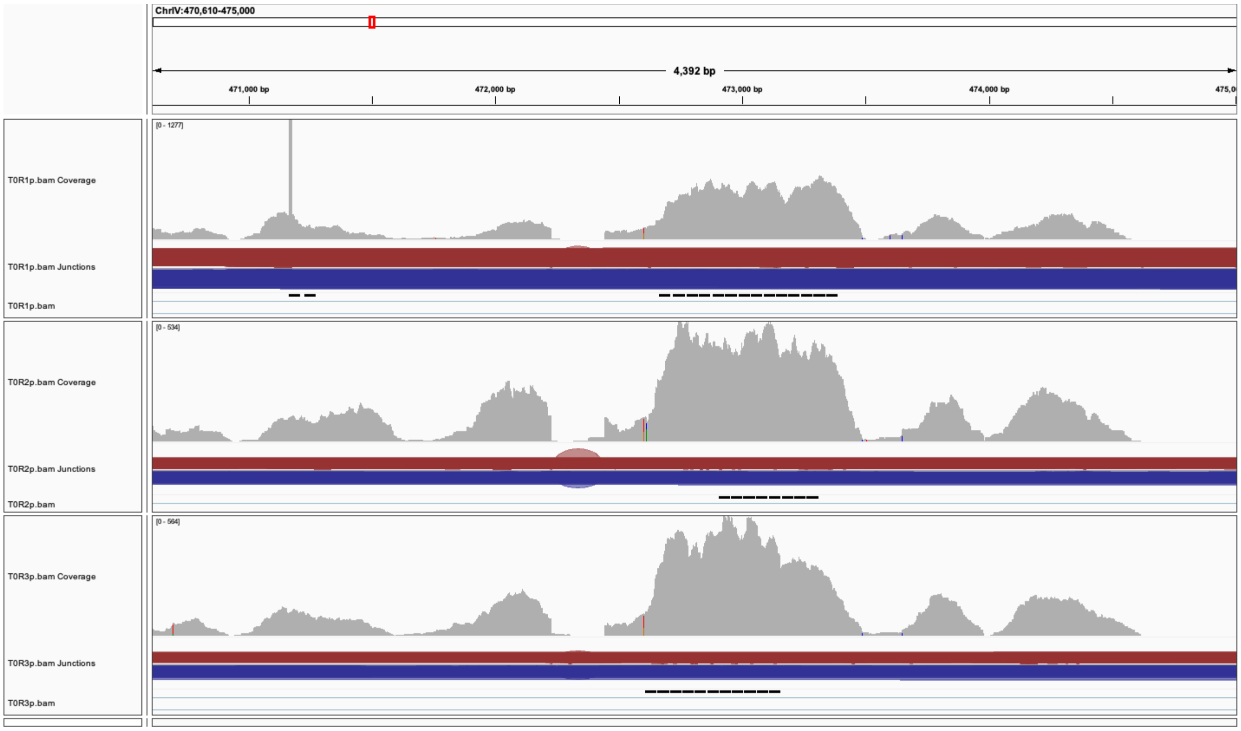

ChrIV:498,221- 509,568 (Central MAT cassette + centromere):

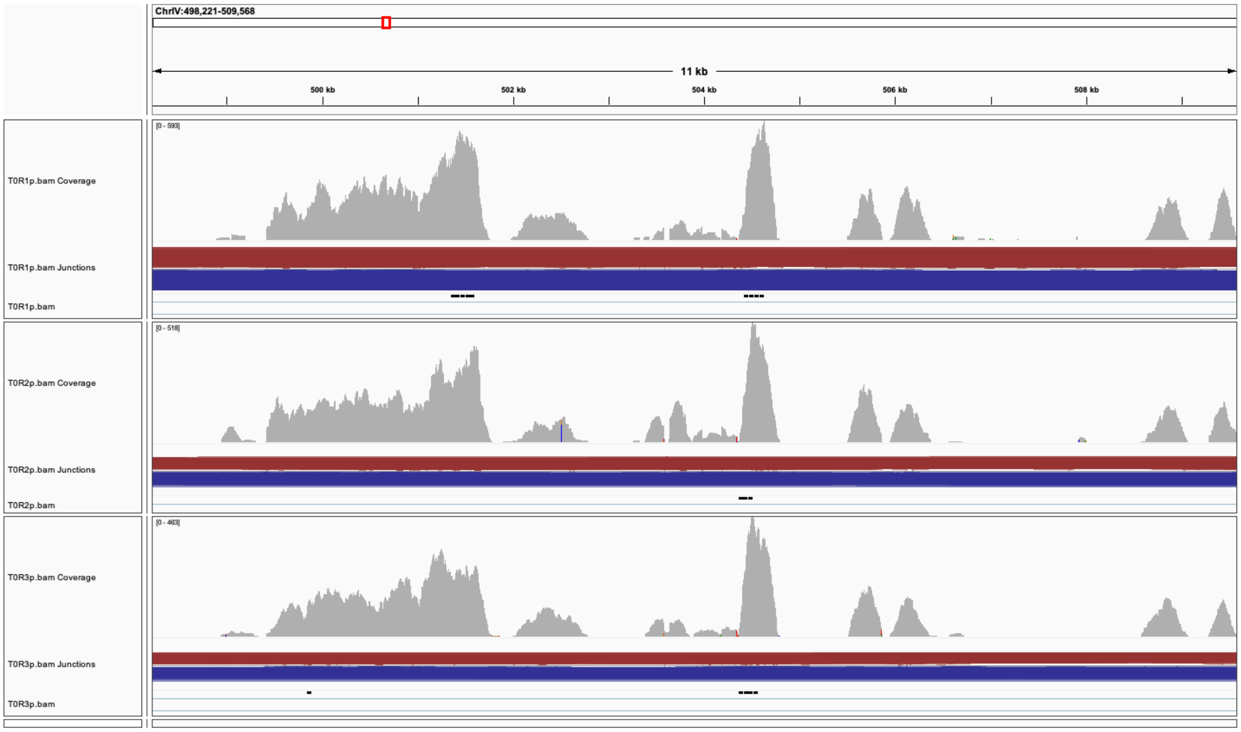

ChrIV:528,044-537,777 (Right MAT cassette):

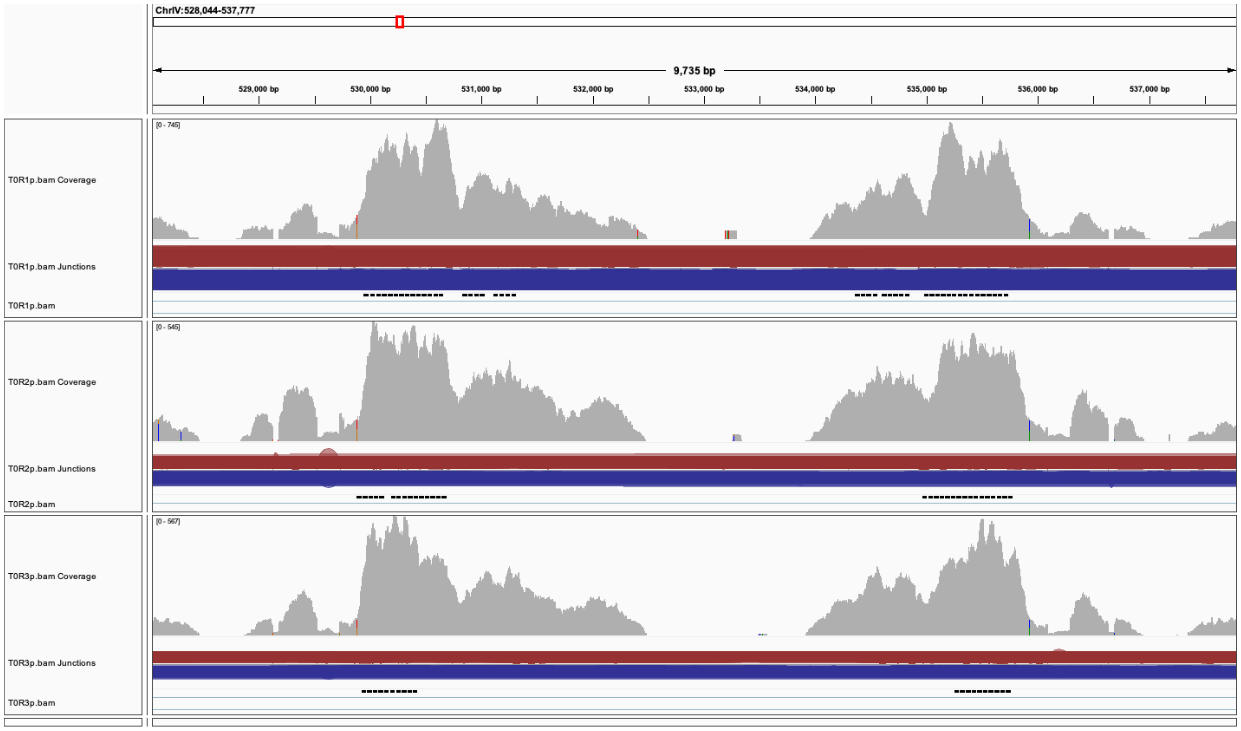

ChrV:2,215,357-2,227,671 (MAT cassette + centromere):

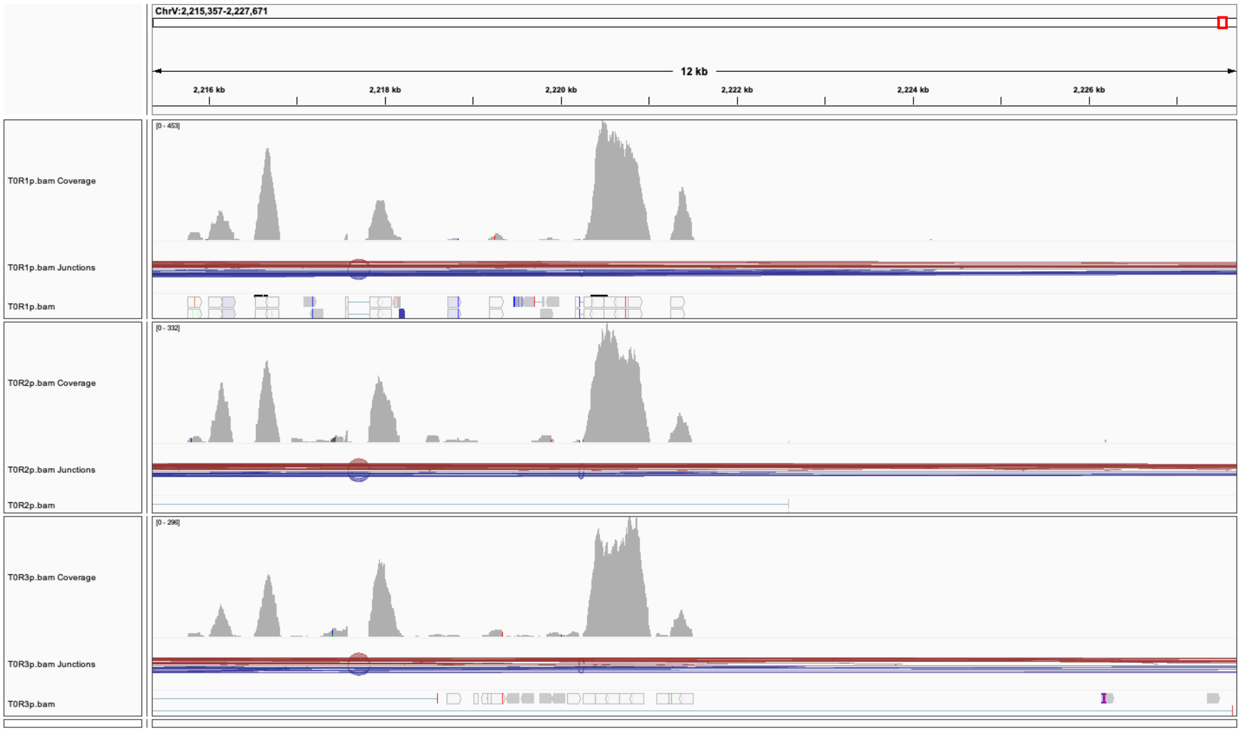

ChrVI:297,472-303,215 (MAT cassette + centromere):

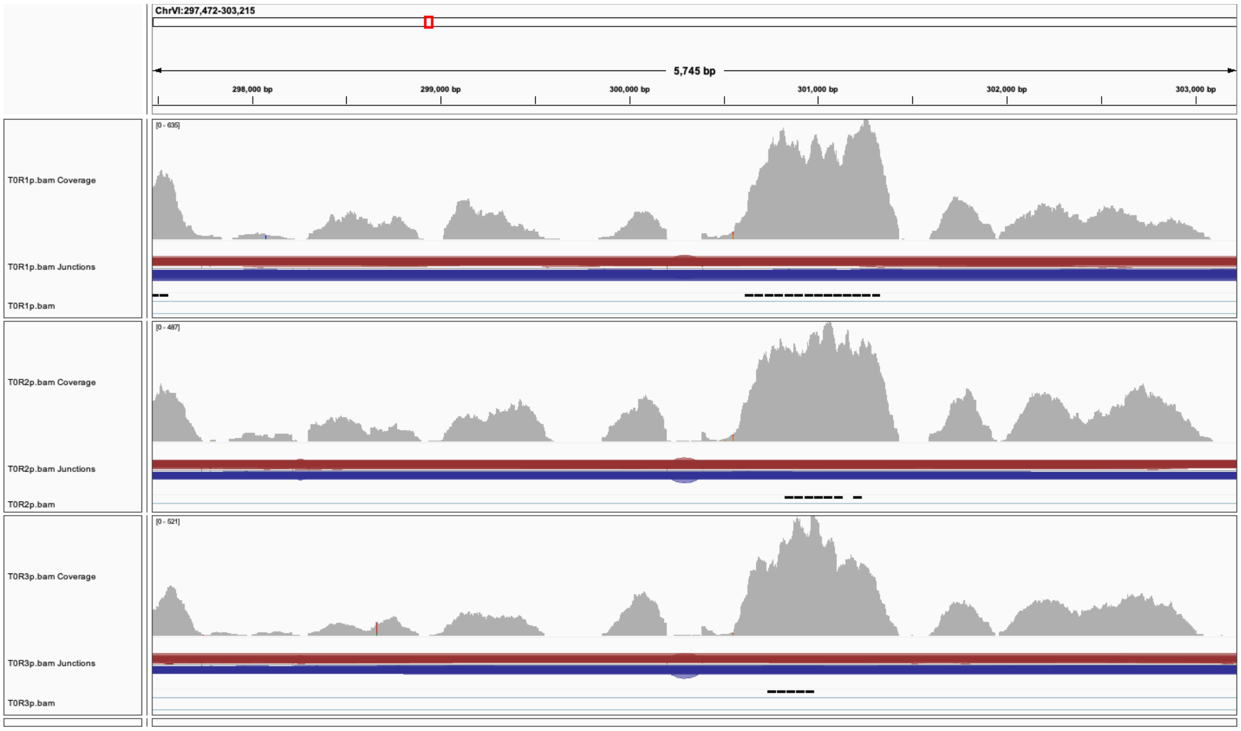

Supplement: jkag067_Supplementary_Data [file jkag067_supplementary_data.zip › Supplementary_File_6_(PDF)_G3-2026-406693.pdf]
