# Supplementary figures and images for: Chromosome-scale genome assembly and characterization of Saccharomycopsis schoenii, a necrotrophic predatory yeast
Source: G3 (Bethesda). 2026 Mar 18;16(5):jkag067. doi: 10.1093/g3journal/jkag067 (PMC13148404; doi:10.1093/g3journal/jkag067)

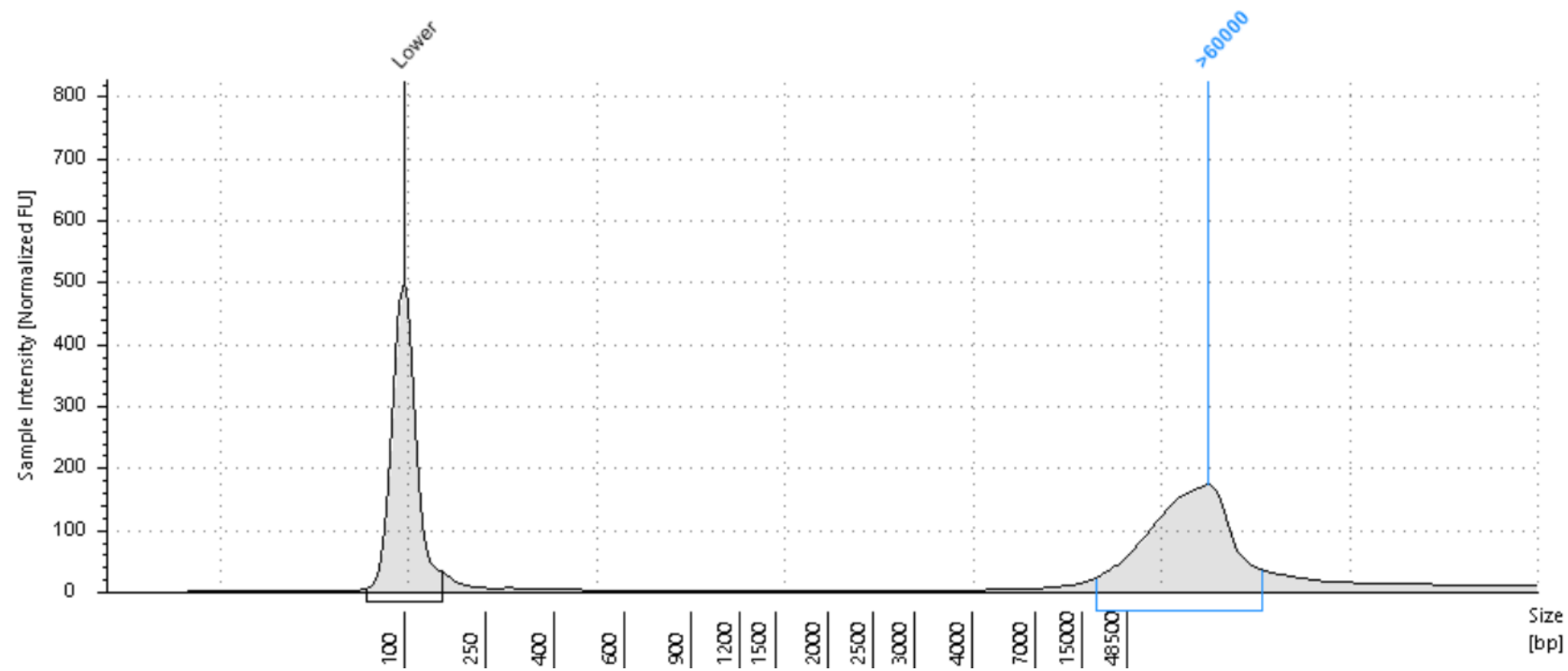

Supplement: jkag067_Supplementary_Data [file jkag067_supplementary_data.zip › Supplementary_Figure_1_G3-2026-406693.pdf]

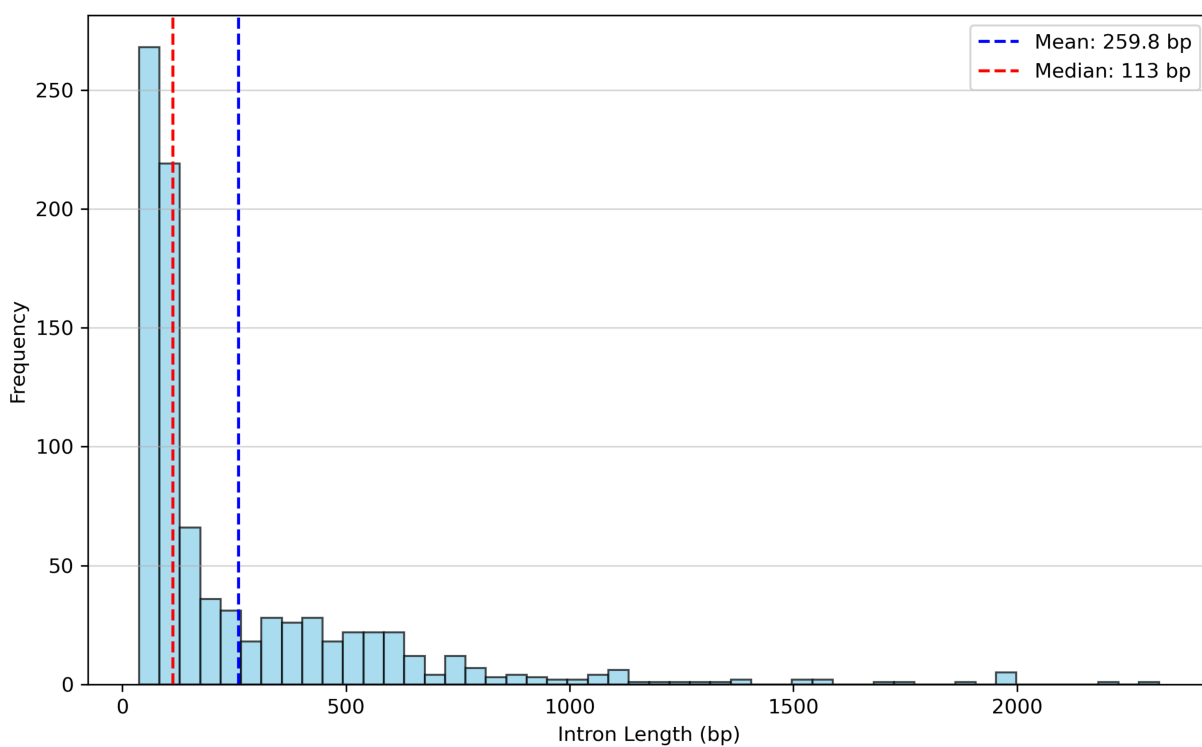

Supplement: jkag067_Supplementary_Data [file jkag067_supplementary_data.zip › Supplementary_Figure_2_G3-2026-406693.pdf]

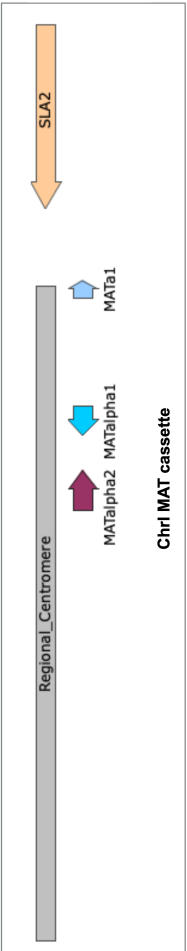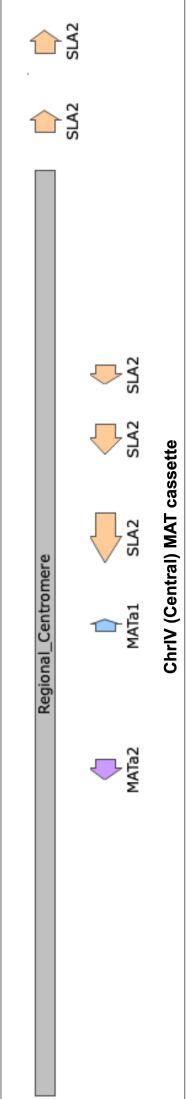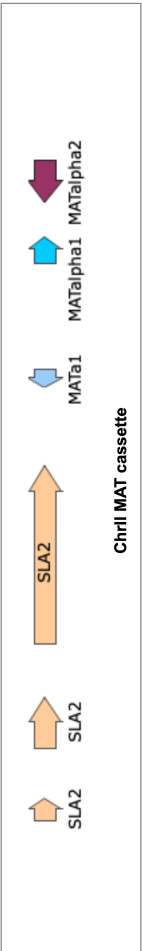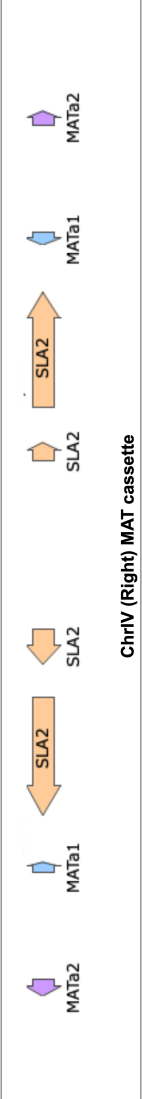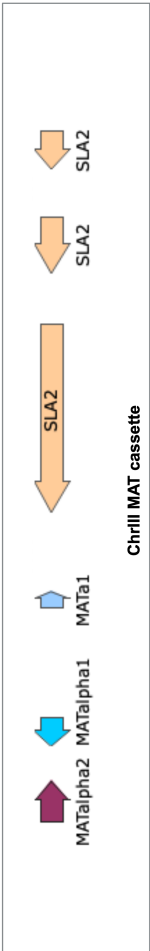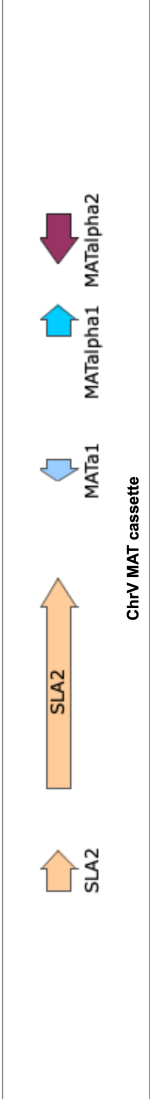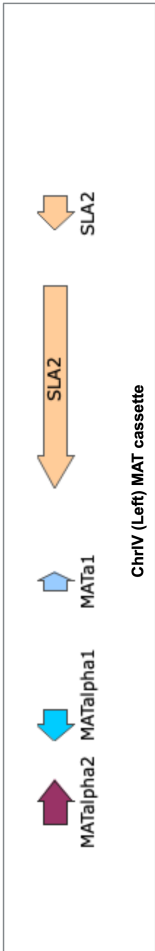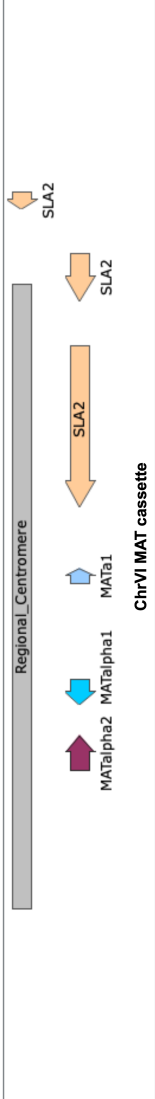

Supplement: jkag067_Supplementary_Data [file jkag067_supplementary_data.zip › Supplementary_Figure_3_G3-2026-406693.pdf]
